# Vascularized Cardiac Tissue Engineering: From Advances in Biofabrication to Translational Applications

**DOI:** 10.1002/advs.74917

**Published:** 2026-03-23

**Authors:** Yang Liu, Zijie Zhang, Hongbin Li, Lino Prados‐Martin, Huanhong Li, Feng Cheng, Xia Tu, Khoon S. Lim, Wanlu Li, Mian Wang

**Affiliations:** ^1^ Shanghai Heart Failure Research Center Shanghai East Hospital School of Medicine Tongji University Shanghai China; ^2^ State Key Laboratory of Cardiology and Medical Innovation Center Institute for Regenerative Medicine Shanghai East Hospital Frontier Science Center for Stem Cell Research School of Life Sciences and Technology Tongji University Shanghai China; ^3^ College of Light Industry and Textile Qiqihar University Qiqihar Heilongjiang China; ^4^ School of Medical Sciences University of Sydney Australia; ^5^ School of Biomedical Engineering Shanghai Jiao Tong University Shanghai China; ^6^ School of Chemistry and Chemical Engineering Harbin Institute of Technology Harbin China

**Keywords:** cardiac cells, cardiac organoids, cardiac tissue engineering, regenerative medicine, vascularized cardiac tissues

## Abstract

The in vitro engineering of vascularized cardiac tissues holds transformative potential for disease modeling, drug screening, and regenerative therapy. However, despite rapid advances in stem cell biology, biomaterials, and biofabrication technologies, the reconstruction of functional, perfusable vasculature within engineered myocardial tissues remains a central and unresolved challenge. In this review, we move beyond a descriptive catalog of available techniques and instead present a process‐oriented framework for understanding vascularized cardiac tissue engineering. By systematically analyzing how cellular components, biomaterial design, and biofabrication strategies collectively govern vascular formation, perfusion stability, and myocardial function, we examine self‐assembly, mold‐casting, 3D bioprinting, and microfluidic approaches, to critically evaluate their respective advantages and trade‐offs under cardiac‐specific physiological constraints. Finally, application prospects of vascularized cardiac tissues in disease modeling and drug testing are discussed, and current limitations and future directions are proposed to accelerate translational impact. By reframing vascularized cardiac tissue engineering as an integrated manufacturing challenge rather than a collection of isolated technologies, this review aims to provide a coherent conceptual guide for advancing functional human cardiac models.

## Graphical abstract

This review presents a process‐oriented framework for vascularized cardiac tissue engineering, highlighting how the coordinated design of cells, biomaterials, and biofabrication strategies enables the functional development or vascularized myocardium for disease modelling and drug discovery.

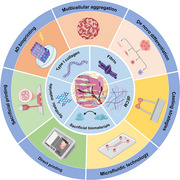

## 1 Introduction

The fabrication of biomimetic in vitro cardiac tissues represents an emerging topic in cardiovascular research, particularly in the context of disease modeling, drug screening, and regenerative therapies [1, 2]. Compared with conventional monolayer cultures or animal models, engineered cardiac tissues provide human‐relevant and controllable microenvironments that enable precise investigation of myocardial physiology and pathology [3, 4, 5]. Importantly, their translational potential extends to therapeutic applications such as myocardial infarction (MI) repair, where intrinsic cardiac regenerative capacity remains limited [1, 6].

Native cardiac tissue is characterized by a highly specialized vascular architecture, which sustains high metabolic demand while accommodating continuous contractile motion and maintaining tight structural coupling with aligned myocardial fibers. The myocardium exhibits high oxygen consumption, requiring a hierarchically organized capillary network tightly integrated with myocardial bundles. In cardiac tissue engineering, failure to recapitulate this vascular complexity, particularly in thick or clinically scalable constructs, leads to diffusion limitations, impaired electromechanical function, and reduced physiological relevance [7, 8, 9]. Accordingly, the in vitro construction of human‐relevant cardiac tissues with perfusable vasculature has always been a key research focus and challenge within the field.

Over the past decade, considerable efforts have been made to engineering vascularized cardiac tissues by integrating advances in stem cell biology, biomaterials science, and biofabrication technologies. Various fabrication strategies, including self‐assembly, mold‐casting, three‐dimensional (3D) bioprinting, and microfluidic perfusion, have been explored to achieve control over tissue geometry, cellular organization, and vascular patterning. The integrating vasculature in engineered cardiac tissues has facilitated the modeling of cardiovascular diseases and increased the efficacy of drug evaluation [10, 11, 12]. While the gap between developmental relevance and engineering controllability remains a key obstacle to constructing human‐relevant cardiac tissues with perfusable vasculature.

In this review, we adopt a process‐driven perspective to examine how vascularized myocardial tissues can be assembled in a manner that recapitulates physiological fidelity with manufacturing feasibility. We first outline the defining vascular characteristics of native cardiac tissue and the key cellular players involved in myocardial vascularization. We then systematically deconstruct major fabrication and assembly paradigms, highlighting their respective strengths, limitations, and trade‐offs under cardiac‐specific physiological constraints. Finally, we discuss unresolved challenges in functional evaluation, scalability, and translational integration, and propose emerging multidisciplinary directions, including dynamic perfusion bioreactors and standardized benchmarking that may guide the next generation of vascularized cardiac tissue models.

## 2 Cell Components for Vascular Engineering of Cardiac Tissues

**FIGURE 1:**
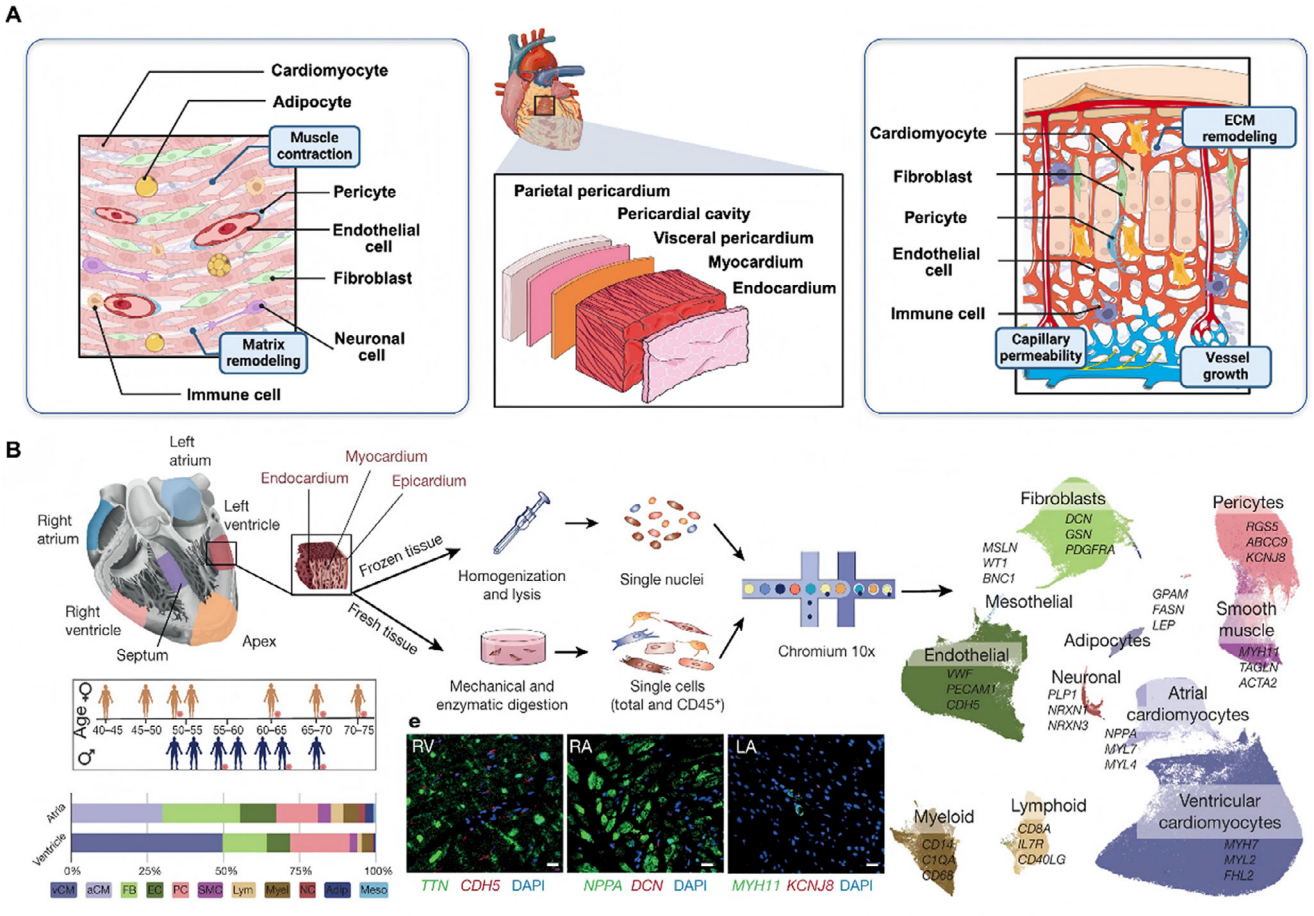
Cellular composition of human cardiac tissues. (A) Schematic illustration depicting the cellular microenvironment of the human myocardium, including key cell types (e.g., CMs, ECs, FBs, immune cells, and pericytes) and their representative physiological roles in cardiac tissue function and homeostasis (This figure was created with BioRender.com). (B) Large‐scale single‐cell and single‐nucleus transcriptomic analyses delineate the cellular landscape of the adult human heart. Scale bars, 20 µm. Reproduced with permission [24]. Copyright 2020, Springer Nature.

Cardiac tissues are primarily composed of cells derived from the mesoderm [13] including cardiomyocytes (CMs), endothelial cell (ECs), cardiac fibroblasts (FBs), mural cells (pericytes and smooth muscle cells (SMCs)), and immune cells (myeloid and lymphoid) [14, 15, 16, 17, 18, 19, 20, 21] (Figure 1A). While myocardium account for approximately 70–85% of the mammalian heart's volume [22], single‐cell transcriptomic analyses reveal that non‐CM populations, particularly stromal and vascular cells, actually dominate in terms of cell number [23]. A comprehensive cellular atlas of the adult human heart showed that atrial tissues contain 30.1% CMs, 24.3% FBs, 17.1% mural cells, 12.2% ECs and 10.4% immune cells. In contrast, ventricular regions (apex, interventricular septum, left and right ventricle) are composed of 49.2% ventricular CMs, 21.2% mural cells, 15.5% FBs, 7.8% ECs and 5.3% immune cells [24] (Figure 1B). This distinct distribution highlights the significance of incorporating defined multicell types in the engineered cardiac tissues to recapitulate the native cellular architecture.

The myocardium exhibits extremely high metabolic demands and relies on a hierarchically organized vascular network to sustain oxygen and nutrient delivery [9, 16, 25, 26, 27]. In engineered cardiac constructs, insufficient vascularization often leads to diffusion limitations, resulting in hypoxia and necrosis in tissues thicker exceeding 100–200 µm in thickness [28]. Therefore, integrating vascular cells has become a key strategy to support CM survival and functional maturation. Indeed, co‐culture with ECs and cardiac FBs not only improves tissue vascularization [29] but also enhances CM maturation through paracrine signaling and extracellular matrix (ECM) remodeling [14]. Collectively, these observations reinforce the need for advanced strategies especially vascularization to bridge the divide between engineered and native myocardium.

### 2.1 Cardiomyocytes as Cornerstones for Cardiac Tissue Engineering

**FIGURE 2:**
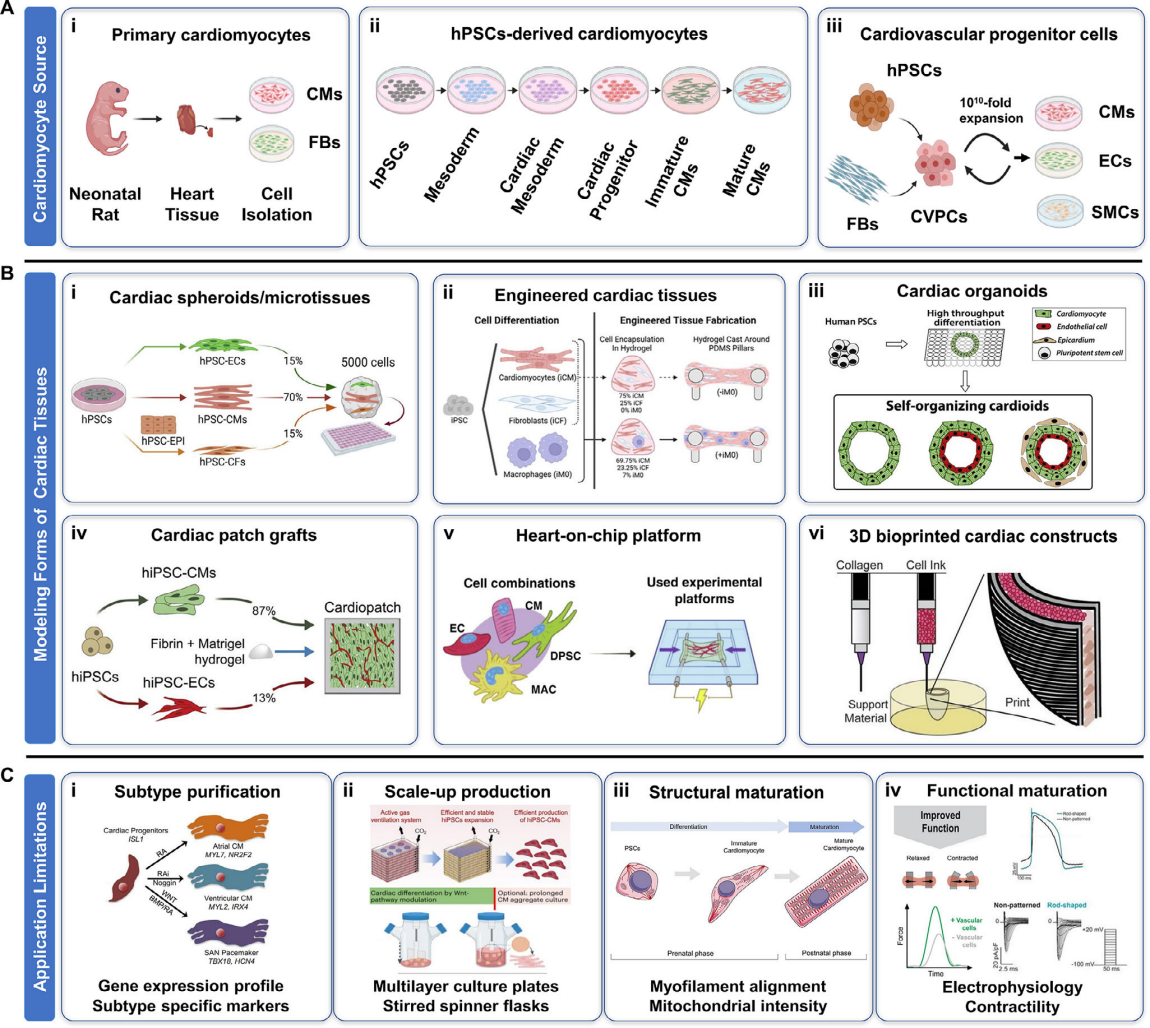
Overview of CMs sources for various forms of cardiac tissue engineering and associated translational challenges. (A) Schematic representation of the main cellular sources and acquiring routes for: (i) primary CMs; (ii) hPSCs‐derived CMs; (iii) human CVPCs (This figure was created with BioRender.com). (B) Representative engineered cardiac tissue modeling utilizing human CMs, including: (i) cardiac spheroids/microtissues by tri‐cellular combinations of cardiac cells. Reproduced with permission [14]. Copyright 2020, Elsevier; (ii) engineered cardiac tissues through mold casting. Reproduced with permission [15]. Copyright 2024, Elsevier; (iii) cardiac organoids via *de novo* differentiation. Reproduced with permission [38]. Copyright 2021, Elsevier; (iv) large‐scale cardiac patch grafts for in vivo transplantation. Reproduced with permission [39]. Copyright 2024, Elsevier; (v) heart‐on‐chip systems supporting perfusable vascular networks. Reproduced with permission [40]. Copyright 2024, Elsevier; (vi) 3D bioprinted cardiac constructs. Reproduced with permission [41]. Copyright 2019, American Association for the Advancement of Science. (C) Key limitations hindering clinical translation of engineered cardiac tissues, including: (i) insufficient differentiation of atrial, ventricular CMs or sinoatrial nodal‐like pacemaker CMs with high purity. Reproduced with permission [42]. Copyright 2020, Alpha Med Press; (ii) challenges in large‐scale expansion. Reproduced with permission [43, 44]. Copyright 2017, Elsevier, and Copyright 2024, Springer Nature; (iii) insufficient structural maturation of CMs like disorganized myofilament and low mitochondrial intensity. Reproduced with permission [45]. Copyright 2021, Frontiers Media; and (iv) poor functional maturation, reflected by electrophysiology and contractile properties. Reproduced with permission [36, 46]. Copyright 2023, Elsevier, and Copyright 2024, Elsevier.

CMs serve as the primary drivers of cardiac contraction and are indispensable to the construction of engineered cardiac tissues [16, 30, 31] (Figure 2). Structurally, CMs contain striated sarcomeres that enable synchronized contraction and gap junctions that support electrical coupling [32, 33]. However, adult mammalian CMs are terminally differentiated and possess limited regenerative capacity, which motivates the development of tissue engineering strategies for cardiac repair [34, 35]. Despite substantial progress, current engineered cardiac tissues often fail to reproduce the dense myofibrillar organization, electrical conduction velocity, and contractile strength observed in native myocardium [36, 37]. Besides, the heterogeneity of CMs subtypes and the limitation regarding human CMs production should also be taking into account (Figure 2C). All these challenges need to be conquered for future use of CMs to construct cardiac tissues.

#### 2.1.1 Primary Cardiomyocytes

In order to engineer vascularized cardiac tissues, former studies used rodent CMs as foundational component for cardiac tissue engineering due to their availability and robust contractile phenotype [47, 48] (Figure 2A‐i). In early studies, these cells were used to form microtissues and cardiac spheroids for disease modeling, investigate intercellular crosstalk under pathological conditions [47], and to achieve vascularization via epicardial implantation [49]. However, physiological and structural differences between rodent and human myocardium limit the translational relevance of these models. Although protocols for isolating human primary CMs have been developed, limited donor availability and technical challenges continue to restrict their widespread use in engineered cardiac tissues [50].

#### 2.1.2 hPSCs‐Derived Cardiomyocytes

The discovery of human pluripotent stem cells (hPSCs), including human induced pluripotent stem cells (hiPSCs), has provided a renewable source of human CMs for cardiac tissue engineering [51, 52]. Differentiation strategies typically mimic embryonic cardiac development through staged modulation of signaling pathways (Figure 2A‐ii). Early protocols relied on embryoid body formation [53], which later evolved into more efficient monolayer‐based differentiation methods [51, 54].

Current approaches primarily utilize two‐dimensional (2D) differentiation systems with significantly improved efficiency, enabling the generation of multiple CM subtypes, including ventricular, atrial, and pacemaker cells [55]. Moreover, large‐scale production of hiPSCs‐derived CMs can be achieved using multilayer culture systems or stirred bioreactors [43, 44] (Figure 2C). These cells have been integrated into various vascularized tissue constructs, including microtissues co‐cultured with ECs [14, 48], cardiopatches with capillary networks [39], organ‐on‐chip models with lumenized vasculature [29], chambered cardiac spheroids assembled via vascular spheroids [10], and 3D bioprinted cardiac constructs with vasculature [56] (Figure 2B).

#### 2.1.3 Cardiovascular Progenitor Cells (CVPCs)

CVPCs are multipotent progenitor cells which have the potential to differentiate into multiple cardiovascular cell types including CMs, ECs and vascular SMCs [57, 58]. CVPCs offer an alternative strategy by simultaneously contributing to the myocardial and vascular compartments (Figure 2A‐iii). Early‐stage CVPCs have been shown to differentiate into CMs and ECs, forming multicellular cardiac tissues that resemble native architecture [59]. Under chemically defined conditions, CVPCs can be captured and expanded from FBs and pluripotent stem cells. Expanded CVPCs robustly differentiate into multiple types of cardiovascular cells and can improve cardiac function after transplantation [60]. In vivo studies demonstrated that engraftment of MESP1^+^ CVPCs could promote myocardial repair through forming CD31^+^ capillaries and promoting angiogenesis in the infarct area [61]. Most recently, Yang and her colleagues reported that co‐seeding CVPCs and CMs onto a decellularized matrix enhanced vascularization and therapeutic outcomes in infarcted hearts [62]. However, the capacity of CVPCs to form mature vasculature autonomously in vivo or within engineered cardiac tissues in vitro remains an open question requiring further investigation.

### 2.2 Vascular Cells for Vascular Reconstruction in Cardiac Tissues

**FIGURE 3:**
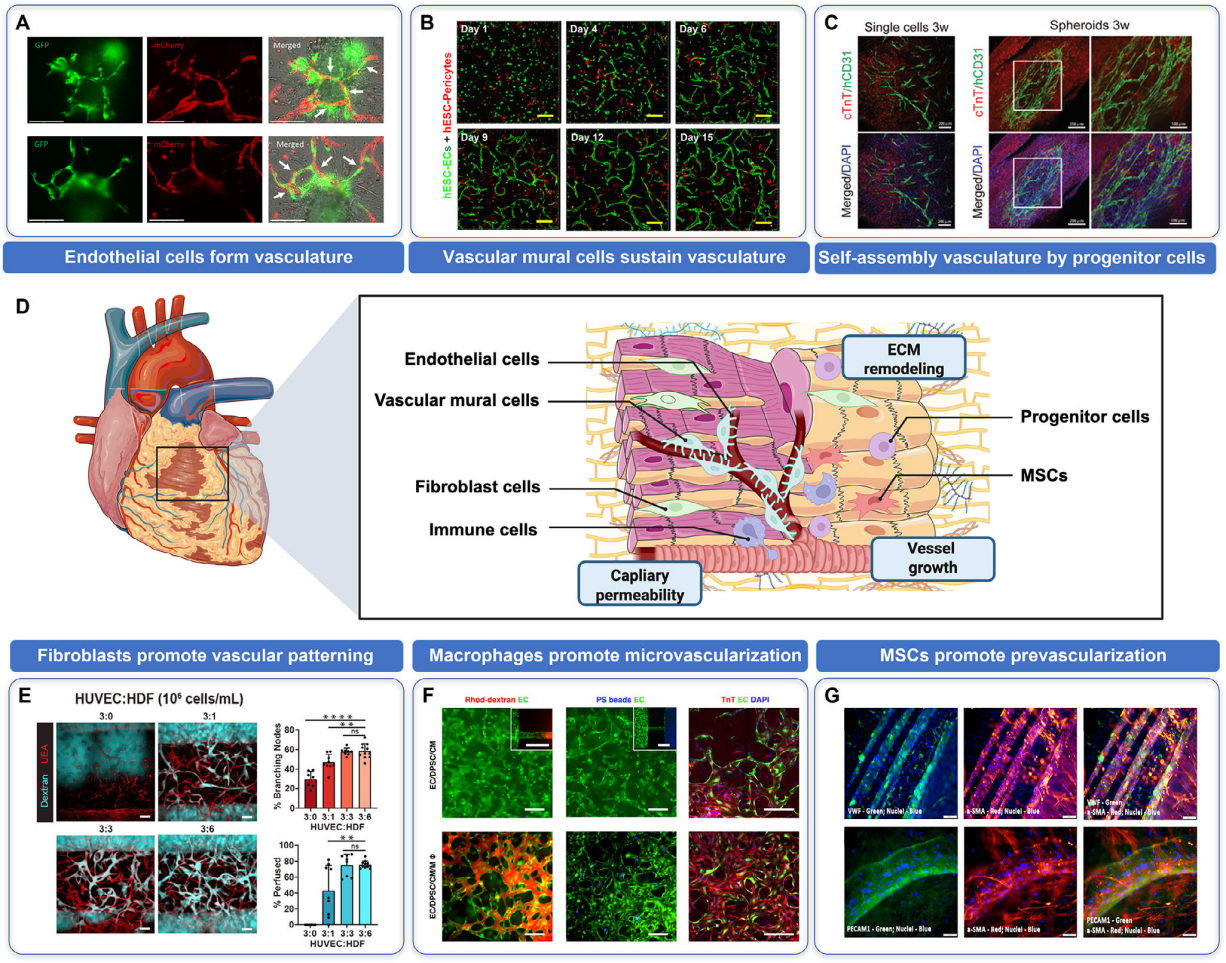
Contribution of vascular and stromal cells in vascularized cardiac tissue engineering. (A) Co‐culture of cardiac microtissues with ECs in chips results in anastomosis and formation of hybrid vessels by interconnection of internal microvascular network (hiPSC‐ECs, GFP) and external vascular network (hiPSC‐ECs, mCherry). Scale bars, 150 µm. Reproduced with permission [29]. Copyright 2023, Elsevier. (B) 3D co‐culture of hESC‐derived ECs and pericytes shows the self‐assembly of ECs into matured microvasular networks with prolonged culture. Scale bars, 200 µm. Reproduced with permission [73]. Copyright 2020, IOP Publishing Ltd. (C) Application of EVCs to enhance vascularization in 3D bioprinted volumetric cardiac tissues. Scale bars, 200 (left and middle) and 100 µm (right). Reproduced with permission [48]. Copyright 2022, Wiley. (D) Schematic diagram summarizing key vascular and stromal cell types and their representative roles in engineered cardiac tissue vascularization (This figure was created with BioRender.com). (E) Endothelial networks (red) were perfused with 500 kDa dextran (cyan) to visualize connected luminal networks of devices fixed on day 7 with varying HUVEC:HDF ratios. Quantification of the percentage of branching network nodes and luminal segments were also shown. Scale bars, 150 µm. Reproduced with permission [74]. Copyright 2020, Wiley. (F) Incorporation of primitive macrophages into engineered cardiac tissues facilitate robust micro‐vascularization. Scale bars, 250 µm. Reproduced with permission [40]. Copyright 2024, Elsevier. (G) In situ vascular structures through co‐culture of ECs and MSCs, highlighting the synergistic effect of stromal‐vascular interaction. Scale bars, 100 µm. Reproduced with permission [75]. Copyright 2017, Frontiers Media.

Compared with most other organs, the coronary circulation is characterized by an exceptionally high capillary density, a feature required to sustain the myocardium's substantial metabolic demand [63]. Cardiac microvasculature is closely aligned with CM bundles and organized hierarchically to support continuous contraction and efficient oxygen delivery [64]. These cardiac‐specific structural and biomechanical features impose unique constraints on the design of vascularized cardiac constructs [65, 66, 67]. From a structure and cellular view, successful vascular engineering requires coordinated contributions from multiple vascular cell populations, including ECs, mural cells (pericytes and smooth muscle cells), and vascular progenitor cells capable of differentiating into vascular lineages [67, 68, 69] (Figure 3D). In engineered vascularized cardiac tissues, recent advances focus on replicating vascular hierarchy based on vascular cells derived from hPSCs, as they can be differentiated toward ECs, pericytes, SMCs or endothelial progenitor cells based on our knowledge about vascular development [70, 71, 72]. This section outlines the roles for each of these cell populations.

#### 2.2.1 Endothelial Cells

ECs possess an intrinsic ability to self‐assemble into vessel‐like networks in 3D hydrogels or extracellular matrices [73, 76]. By leveraging this endothelial patterning properties of ECs, self‐organized vascular networks have been generated within different 3D multicellular constructs, including vascularized skeletal muscle tissue [77], vascularized brain organoid [78, 79], vascularized kidney tissue [80, 81] and so on. Among them, human umbilical vein endothelial cells (HUVECs) are most commonly used for vascularization in cardiac tissue mimetics [76, 82] and engineered cardiac patches [83]. A recent report showed that co‐culturing HUVECs with hiPSCs‐derived CMs in a composite hydrogel of fibrin (5 mg/mL) and collagen I (0.2 mg/mL) led to the formation of microvascular lumens and myocardial clusters at the millimeter scale, owing to the matrix's low stiffness and hyperelasticity [82]. In parallel, engineered microchannels with defined geometries have been shown to guide EC alignment and morphogenesis, enabling the formation of patent microvessels, which sprouted in accordance with the channel orientation [84]. However, it has been confirmed in mice and humans that endothelium from different tissues and vessel types is highly specific to meet distinct functional demands, which cannot be achieved by fixed‐source HUVECs [85, 86, 87]. To overcome this limitation, human embryonic stem cells (hESCs)‐derived ECs have been shown to form perfusable microvascular constructs in patterned microchannels embedded in collagen matrix, which effectively support hiPSCs‐derived CMs in engineered cardiac tissues after implantation [88].

In microphysiological systems, vascularized cardiac tissues can also be generated by co‐culturing hiPSCs‐derived ECs and CMs within engineered hydrogels or organ‐on‐chip platforms, producing interconnected vascular networks that enhance tissue maturation and vascular integration [29, 39] (Figure 3A). Moving forward, the generation of physiologically adaptable ECs, such as through ETV2 reactivation in 3D cultures enriched with basement membrane components (e.g., laminin, entactin, and type IV collagen), which may further improve the integration, stability, and function of vascular networks in engineered cardiac tissues [89]. In addition to intrinsic endothelial programming, endothelial self‐assembly is strongly influenced by supportive cellular interactions and paracrine signaling from the surrounding microenvironment. For example, incorporating synthetic cells engineered to secrete recombinant human bFGF into EC‐laden constructs promotes the formation and stabilization of 3D vascular networks [90].

#### 2.2.2 Vascular Mural Cells

While ECs are essential for initiating vasculature formation, they are insufficient alone to establish stable, functional vessels [91, 92]. Vessels composed solely of ECs tend to be unstable and prone to regression over time [93]. To ensure long‐term vascular integrity, the support of mural cells, specially pericytes and SMCs are of importance in structural support, vessel maturation, and contractile regulation of micro‐ and small‐diameter vessels [94]. Recent studies have demonstrated that co‐culturing hPSCs‐derived ECs and pericytes within fibrin‐based hydrogels facilitates the formation of stable, lumenized microvasculature that remains stable for at least 3 weeks [73] (Figure 3B). Voges et al. reported that addition of vascular cells in human cardiac organoids significantly enhances tissue maturation through paracrine mechanisms, with NG2^+^ pericytes found to surround the endothelial networks [46]. Similarly, PECAM^+^ and PDGFRβ^+^ (pericytes‐specific marker) microvessel networks were observed enveloping the surface of structurally and functionally relevant heart organoids [95] (Figure 3B). Furthermore, stem cell‐derived blood vessel organoids embedded in collagen I‐Matrigel gels have recapitulated capillary‐like architectures composed of ECs, pericytes, and mesenchymal stem‐like cells [96]. More recently, dynamic hydrogel culture systems have been shown to enhance angiogenic activity and promote differentiation toward arteriole‐like vessels containing SMCs, indicating the importance of biomechanical cues in mural cell specification and vessel formation [97].

#### 2.2.3 Vascular Progenitor Cells

Vascular progenitor cells, including endothelial progenitor cells or EVCs, provide another promising cellular source for constructing vascularized cardiac tissues. Endothelial progenitor cells, which serve as precursors of ECs, can be derived from hiPSCs [72]. These cells possess the capacity to proliferate and self‐organize into branched tubular structures, forming capillary‐like vascular tubes or networks ranging from 10 to 100 µm [98]. Our previous work demonstrated that CD34^+^ EVCs can function as vascular seeds for microvascular network formation in both cardiac spheroids and 3D‐bioprinted cardiac patches [48] (Figure 3C). In parallel, hiPSCs‐derived vascular spheres composing of ECs and CD34^+^ progenitors have been incorporated with CMs to generate chambered, vascularized constructs termed vaschamcardioids [10].

Despite these advances, achieving long‐term perfusion and physiologically mature vascular networks remains a major challenge in engineered cardiac tissues. To address this limitation, alternative approaches incorporating pre‐formed vascular units have recently been explored. For example, primary microvessel fragments isolated from adipose tissue retain endothelial lumens and perivascular cell coverage and can rapidly integrate into engineered cardiac constructs, promoting vascular network formation and supporting CM survival without the need for exogenous growth factors [99, 100].

### 2.3 Cardiac Stromal Cells for Vascular Formation

In addition to CMs and vascular cells, stromal cell populations play essential roles in regulating myocardial homeostasis and vascular development [101]. Cardiac stromal cells include FBs, immune cells, and other resident supporting cells that collectively modulate ECM remodeling, intercellular communication, and tissue repair processes [102]. Increasing evidence indicates that these stromal populations actively participate in vascular formation and tissue integration within engineered cardiac tissues [14, 40]. From an engineering perspective, incorporation of cardiac stromal cells into vascularized cardiac tissues is pivotal for promoting vascular formation, immune regulation, and functional tissue maturation (Figure 3D). Although challenges persist, such as precisely controlling cardiac FBs activity to avoid pathological fibrosis, the active and multifaceted functions of stromal cells clearly transcend passive support.

#### 2.3.1 Cardiac Fibroblasts

Cardiac FBs represent approximately 15.5% of the cellular population in human ventricular myocardium and are key regulators of ECM synthesis and remodeling [24]. In engineered cardiac tissues, FBs contribute to vascular stabilization and tissue maturation through paracrine signaling and ECM deposition. For example, vascularized cardiac constructs composed of human CMs, ECs, and embryonic FBs showed reduced EC apoptosis, increased EC proliferation, and improved vessel stability [102, 103]. In 3D vascularized cardiac tissue mimetics containing cardiac FBs, transforming growth factor‐β (TGF‐β) stimulation significantly increased collagen deposition and promoted endothelial‐to‐mesenchymal transition, as demonstrated by Krishnan et al. [47]. Beyond their structural roles, cardiac FBs also facilitate CM alignment and electrophysiological maturation [104], partly through fibronectin‐rich ECM deposition and electrical coupling with adjacent CMs [74] (Figure 3E). When CMs, ECs and FB were physically separated, the significant and distinct regulatory influence on hiPSCs‐derived CM electrophysiology observed in CM‐EC‐FB co‐culture system were lost [105]. Consistently, co‐culture with cardiac FBs enhances the molecular and functional maturation of hiPSCs‐derived CMs in both scaffold‐based and scaffold‐free 3D cardiac microtissues [14, 106]. Prevascularized cardiac microtissues have also been constructed by combining hiPSCs‐derived CMs, ECs and FBs in a ratio of 70:15:15 [29], yielding improved structural and vascular integration.

Epicardial‐derived cells represent another stromal source contributing to vascular development. These cells undergo epithelial‐to‐mesenchymal transition (EMT) and can differentiate into fibroblast or mural cell lineages under appropriate signaling conditions [107]. Meanwhile, epicardial cells have shown to enhance angiogenesis and lymphangiogenesis following intramyocardial injection in vivo [108]. However, their functional contribution to vasculature formation in engineered cardiac tissues remains to be fully elucidated [106].

#### 2.3.2 Immune Cells

Immune cells play vital roles in tissue surveillance, homeostasis, and regeneration [109, 110]. In the cardiac microenvironment, resident macrophages and regulatory T cells (Tregs) have emerged as key contributors to vascular development and tissue remodeling. Cardiac resident macrophages were reported to stimulate angiogenesis and inhibit fibrosis during early stages of hypertrophy development [111]. Tregs are key immune regulators that have shown promise in enhancing cardiac repair post‐MI [112]. Most recently, an in vivo study showed that Tregs mediates repair by modulating monocytes/macrophages, and mice receiving Tregs had increased blood vessel density in the infarct zone by immunostaining for the EC marker CD31 4 weeks post‐treatment [113]. The inclusion of resident cardiac macrophages enhanced contractile force production and cardiac function in hiPSCs‐derived engineered cardiac tissues models [15]. Notably, hPSCs‐derived primitive yolk‐sac‐like macrophages incorporation profoundly impacted the functionality and perfusability of microvascularized cardiac tissues up to 2 weeks of culture [40] (Figure 3F). In addition, a newly research reported heart‐forming organoids recapitulating aspects of heart, vasculature and foregut co‐development, which resembles aspects of haematopoietic development and blood‐generating [114].

#### 2.3.3 Mesenchymal Stem Cells (MSCs)

As a type of adult multipotent stem cells, MSCs can be isolated from various tissues including the umbilical cord, bone marrow, and adipose tissue. These cells exhibit multilineage differentiation potential, giving rise to osteoblasts, chondrocytes, adipocytes, and SMCs [115]. MSCs are widely regarded in regenerative medicine due to their broad availability, ease of isolation, and low immunogenicity [116]. In the context of vascularized cardiac tissues, MSCs were reported to facilitate endothelial differentiation and vascular formation, assist long‐term survival and functional maintenance primarily through the secretion of paracrine factors [117, 118]. For example, human microvascular ECs and MSCs were cocultured onto a 3D collagen cell carrier for 7 days under vasculogenic culture conditions to generate prevascularized scaffold. And subsequent co‐culture with embryonic CMs led to the formation of vascularized cardiac tissues under myogenic conditions [75, 119] (Figure 3G). Beyond angiogenesis, mesenchymal or stromal cells also contribute to vascular stability and can promote self‐assembly of microvascular networks [120]. However, despite these advantages, MSC‐based strategies face challenges in achieving functional alignment and synchronized integration between newly formed vessels and CM networks, which remains a critical barrier to fully biomimetic cardiac tissue construction [121].

## 3 Biomaterials Used in Vascular Scaffold Fabrication

### 3.1 Protein‐based Biomaterials for Vascularized Cardiac Tissue Engineering

#### 3.1.1 Type I Collagen

**FIGURE 4:**
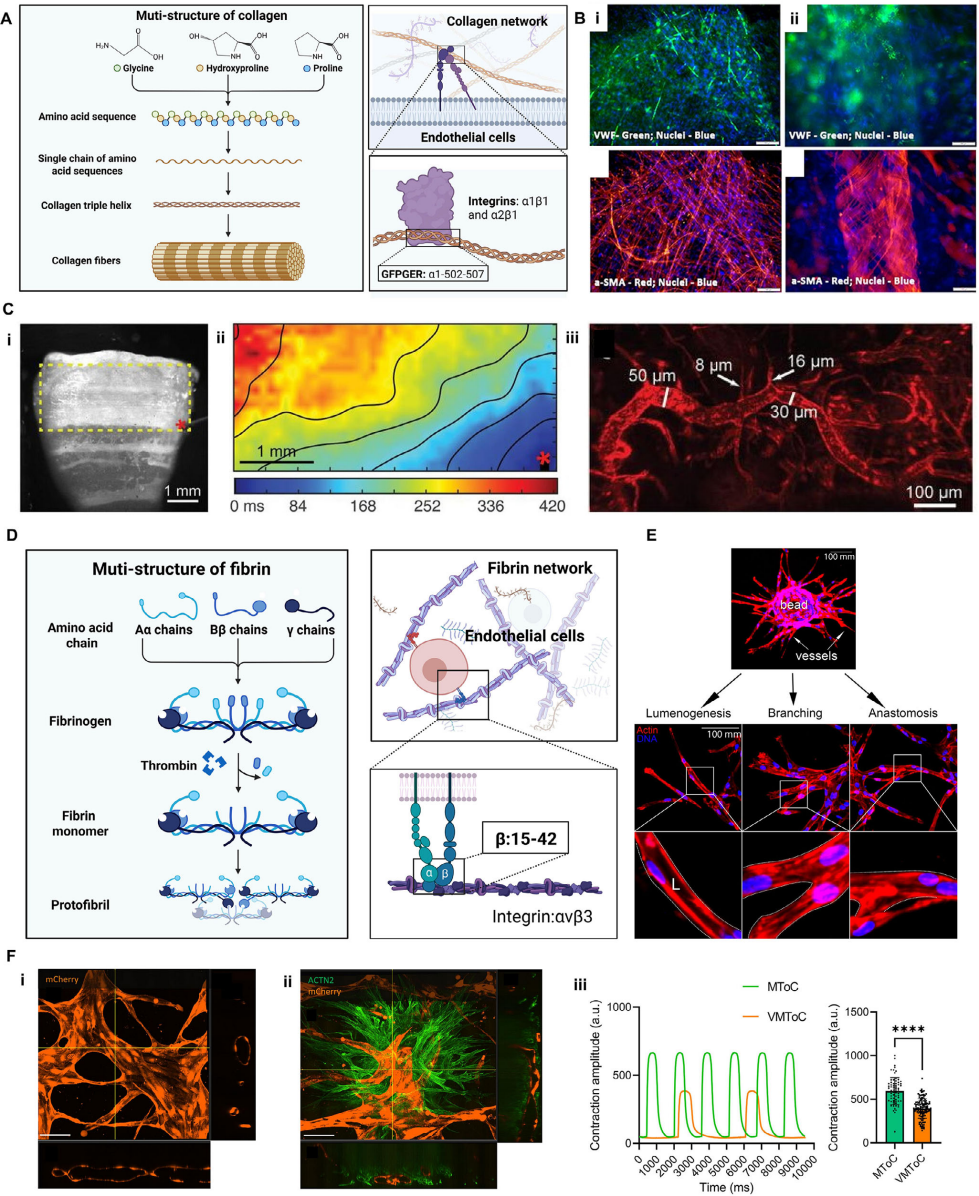
Collagen and fibrin used for vascularized cardiac tissues fabrication. (A) Schematic illustration of multi‐scale hierarchical structure of collagen and its interactions with ECs, emphasizing its role in supporting vascular morphogenesis (This figure was created with BioRender.com). (B) Immunofluorescence of vascular structures formed by hCMVECs / hMSCs co‐culture within collagen bioink. (i) Intricately branched microvasculature expressing both endothelial and SMC markers; (ii) Formation of small‐ and medium‐sized muscular arteries within collagen matrix. Scale bars, 100 µm. Reproduced with permission [75]. Copyright 2017, Frontiers Media. (C) FRESH‐printed collagen ventricle constructs. (i) Side view of FRESH printed collagen‐based ventricle stained with calcium‐sensitive dye. Scale bars, 1 mm; (ii) Calcium mapping of the subregion. Scale bars, 1 mm; iii) Evidence of in vivo vascular infiltration within implanted FRESH‐printed collagen disk. Scale bar, 100 µm. Reproduced with permission [41]. Copyright 2019, American Association for the Advancement of Science. (D) Schematic illustration of fibrin's hierarchical structure and its interactions with ECs (This figure was created with BioRender.com). (E) Fibrin bead assay modeling key features of angiogenesis in vitro. Left: representative image of embedded fibrin bead after 4 days of growth. Right: images depicting key angiogenic events including lumenogenesis, vessel branching, and anastomosis. Scale bars, 100 mm. Reproduced with permission [129]. Copyright 2021, Wiley. (F) Vascularized cardiac microtissue on chip. Representative confocal images of microvascular network in vessel‐on‐chip (i), vascularized cardiac MT on chip (ii) showing hiPSCs‐ECs (orange, mCherry) and hiPSCs‐CMs (green, ACTN2), iii) Representative beating traces and quantification of contraction duration. Scale bars, 100 µm. Reproduced with permission [29]. Copyright 2023, Cell Press.

Type I collagen is one of the most ubiquitous and versatile natural biomaterials for vascularized tissue engineering due to its abundance in native extracellular matrices and its ability to support both mechanical integrity and cell‐matrix interactions [106, 122]. Early work by Davis et al. demonstrated that ECs can invade and form capillary‐like branching structures within collagen matrices, a process further enhanced by angiogenic cues such as bFGF and VEGF [123, 124]. At the molecular level, this behavior depends on specific collagen motifs, such as the glycine‐phenylalanine‐proline‐glycine‐glutamic acid‐arginine (GFPGER) sequence, which interacts with integrins α1β1 and α2β1 expressed on EC surfaces [125, 126] (Figure 4A). In addition to ligand‐receptor interactions, the physical properties of the collagen matrix, particularly its concentration, strongly influence endothelial behavior [127, 128]. These observations established collagen as a permissive microenvironment for endothelial morphogenesis and vascular assembly.

Building on these principles, collagen has been used to create vascularized cardiac tissues and has been shown to promote both blood vessel growth and CM maturation. Further improvements were investigated by involving supportive stromal populations, such as FBs or human mesenchymal stem cells (hMSCs), which modulate matrix remodeling and paracrine signaling within the collagen scaffold [75, 130] (Figure 4B). Beyond static hydrogels, collagen has also been incorporated into dynamic platforms such as organ‐on‐a‐chip systems and 3D bioprinting (Figure 4C), where it supports multiscale vascular organization under perfusable or mechanically active conditions [41, 88].

#### 3.1.2 Fibrin

Fibrin, the primary structural component of blood clots, has been extensively adopted as a biomaterial for wound healing and angiogenesis modeling (Figure 4E) [131, 132]. Its microporous and nanofibrous architecture provides a substrate for cell adhesion and migration [133]. Fibrin gels are formed through the enzymatic conversion of fibrinogen by thrombin (Figure 4D), and factor XIIIa is often applied to covalently cross‐link fibrin, thereby improving network stability [134]. An advantage of fibrin‐based matrices is the tunability of their mechanical properties, which can be adjusted by varying the fibrinogen‐to‐thrombin ratio [135], while degradability can be temporally controlled using protease inhibitors such as aprotinin [136, 137] or 6‐aminocaproic acid [138]. Similar to collagen, fibrin concentration strongly influenced endothelial tube formation [139]. As a result, most studies employ fibrin at concentrations of 5–10 mg/mL. Fibrin primarily interacts with ECs through αvβ3 integrins (Figure 4D), which are abundantly expressed on human ECs and mediate attachment to fibrinogen, fibronectin, and vitronectin [140].

Beyond receptor‐mediated signaling, the physical characteristics of fibrin matrices significantly influence EC behavior. For example, Hinsbergh and colleagues demonstrated that altering fibrin properties by adjusting pH, incorporating heparin, or using mutant fibrinogen, significantly affected angiogenic outcomes [141]. Furthermore, fibrin serves as a reservoir for pro‐angiogenic growth factors, including VEGF and bFGF, thereby enhancing endothelial proliferation and vascular morphogenesis. These features make fibrin an attractive biomaterial candidate for engineering vascularized cardiac tissues. For instance, Arslan et al. co‐cultured ECs with cardiac organoids within fibrin‐based microfluidic systems and presented interconnected vascular networks both inside and surrounding the vascularized cardiac microtissue, which maintained rhythmic contractions throughout the experiment (Figure 4F) [29]. Similarly, pre‐vascularized stromal patches generated using fibrin scaffolds have shown enhanced vascular network formation and improved CM survival [142]. Beyond hydrogel formulations, electrospun fibrin microfibers have also been utilized to enhance mechanical robustness and guide cellular alignment [143]. Such platforms support both cardiac electrophysiology and vascular network formation, highlighting the versatility of fibrin as a biomaterial for engineering vascularized cardiac tissues.

#### 3.1.3 Decellularized Extracellular Matrix (dECM)

**FIGURE 5:**
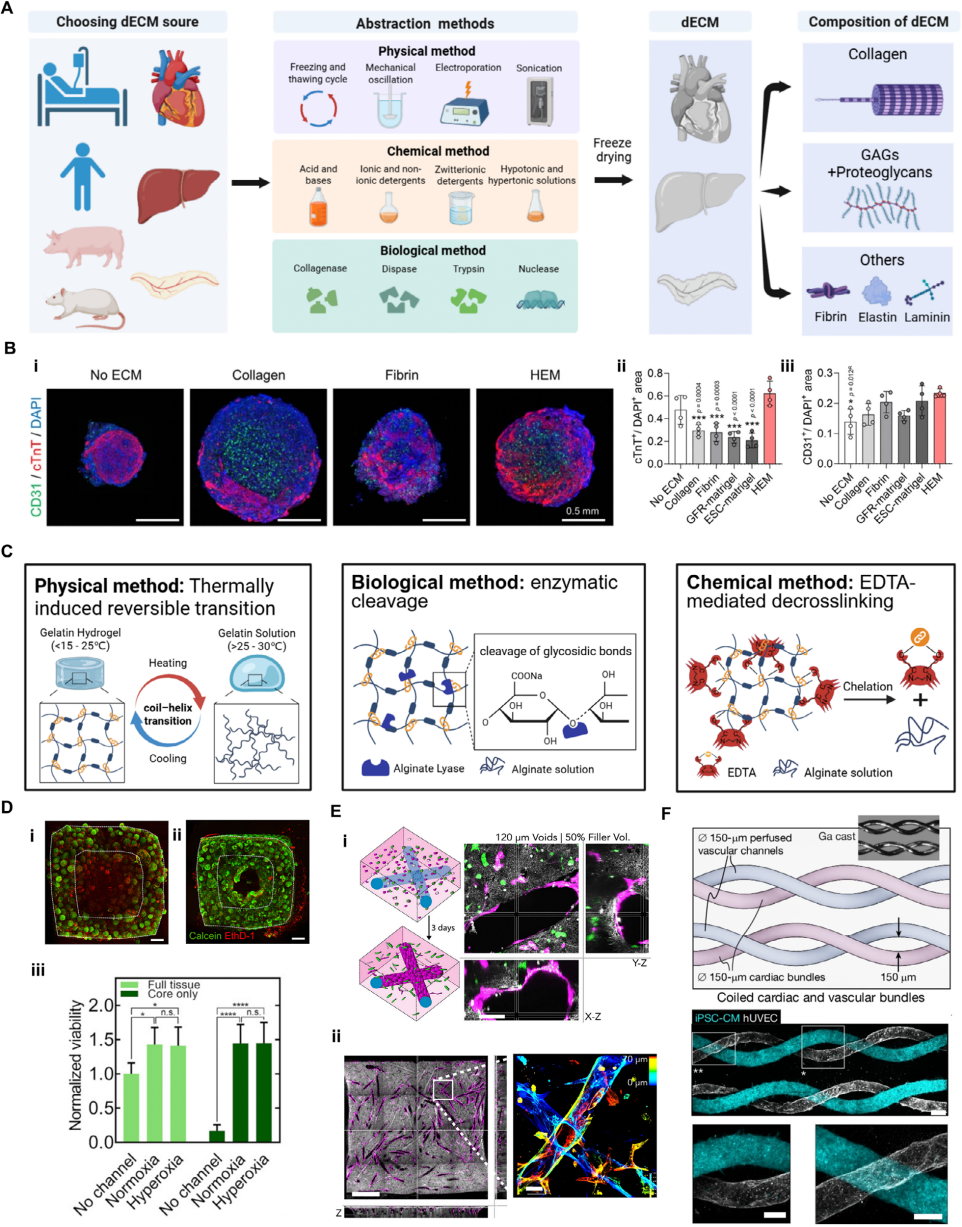
Utilization of dECM and sacrificial biomaterials in the fabrication of vascularized cardiac tissues. (A) Schematic diagram illustrating the source tissues, decellularization methods, and main biochemical components of dECM used for cardiac tissue engineering (This figure was created with BioRender.com). (B) (i) Representative immunofluorescent images and quantification of the areas positive for (ii) cardiomyocyte (CM; cTnT) and (iii) endothelial cell (EC; CD31) markers in each group of cardiac tissues. Scale bars = 0.5 mm. Reproduced with permission [8]. Copyright 2024, Springer Nature. (C) Schematic diagram of sacrificial biomaterials’ cleavable pathway (This figure was created with BioRender.com). (D) Viability analysis of cardiac tissues following 12 h of culture. (i) Without channels, (ii) with channels perfused using hyper‐oxygenated (95% O_2_) medium, and (iii) quantification demonstrating enhanced cell viability with perfusion. Scale bars, 500 µm. Reproduced with permission [159]. Copyright 2019, American Association for the Advancement of Science. (E‐i) Tri‐culture of CMs, human dermal fibroblasts, and HUVECs in a sacrificially templated scaffold. (ii) ECs migrate and form monolayers along perfusable voids. Scale bar, 1 mm (top), 50 µm (bottom left), 50 µm (bottom right). Reproduced with permission [160]. Copyright 2024, Cell press. F) Hierarchical vascular trees and cell‐dense structures with proximal vasculature using ESCAPE method. (i) 3D branching vascular networks visualized by cell nuclei color‐coded of z‐depth. (ii) Tile scans and close‐up images of the coiled section showing all cells, hUVECs (UEA lectin) and iPSCs‐CMs (TTN‐GFP). Scale bars, 200 µm (i), 100 µm (ii). Reproduced with permission [161]. Copyright 2024, Springer Nature.

The native ECM constitutes a structurally and biochemically complex network whose composition and organization vary across different tissues [144, 145]. This tissue specificity presents a challenge in tissue engineering, as reproducing native cellular microenvironments using a single synthetic or naturally derived biomaterial remains difficult. As a result, dECM has gained attention as an approach for preserving tissue‐specific biochemical cues that regulate cell behavior, differentiation, and maturation [146, 147, 148]. dECM is produced by removing cellular components from native tissues through a combination of physical [149, 150, 151], chemical [152, 153, 154], and enzymatic processes [155, 156], while retaining a complex matrix enriched in tissue‐derived structural proteins, growth factors, and other bioactive molecules (Figure 5A). For engineering vascularized cardiac tissues, heart‐derived dECM (h‐dECM) provides the most direct and organ‐specific microenvironment [157]. Al‐Hejailan et al. validated the compatibility of the resulting h‐dECM with ECs, CMs, and perivascular cells [158]. Building upon this, Min et al. used left‐ventricle‐derived h‐dECM as material for prevascularized cardiac constructs, which enhanced cardiac progenitor cell maturation and promoted vascular network formation compared with conventional bioinks (Figure 5B) [8].

Beyond the heart, the omentum offers another valuable source of dECM, owing to its high vascular density and availability from human donors [162]. Unlike the cardiac tissues, which have limited accessibility, the omentum can be harvested with relatively minimal invasiveness. Researchers used healthy or even patient‐specific omental tissue to fabricate personalized dECM hydrogels [56, 163]. These hydrogels then be used to fabricate perfusable cardiac tissue through different methods. The good vascularization results all demonstrate the great potential of this particular material in the construction of vascularized tissues.

#### 3.1.4 Gelatin and Gelatin‐Derived Material

Gelatin, a denatured derivative of collagen, is a biocompatible polypeptide mixture that retains cell‐adhesive Arg‐Gly‐Asp (RGD) motifs and protease‐sensitive sites, making it a favorable scaffold material in tissue engineering [164, 165]. It can be cross‐linked via chemical (e.g., glutaraldehyde) or enzymatic methods (e.g., microbial transglutaminase, mTG) to improve mechanical stability [166, 167]. When freeze‐dried, gelatin forms a porous architecture that facilitates EC migration and supports angiogenesis. Leveraging this property, Pagliari et al. [168] developed pre‐vascularized 3D cardiac biosubstitutes using gelatin scaffolds, demonstrating enhanced vascular network formation.

To improve its mechanical tunability and printability, gelatin has been chemically modified to create GelMA, which incorporates methacrylamide groups enabling covalent cross‐linking via photopolymerization [169, 170]. GelMA retains the bioactive features of gelatin, including RGD sequences, but offers superior structural fidelity and compatibility with advanced biomanufacturing techniques such as 3D bioprinting [171, 172]. For instance, Kupfer et al. utilized GelMA to 3D print electromechanically functional, chambered cardiac organoids composed of contiguous muscle tissue, illustrating its potential for constructing vascularized and functional myocardial models [173].

### 3.2 Sacrificial Biomaterials for Vascularized Cardiac Tissue Engineering

The sacrificial molding strategy has emerged as a powerful and versatile approach to fabricate perfusable vascular networks within hydrogel‐based cardiac tissue constructs [174]. This approach involves embedding a removable template that can be selectively dissolved to create interconnected luminal channels resembling native vasculature. According to the mechanism of sacrifice, we categorized these materials into thermoresponsive materials, chemically or biologically sensitive materials, and others (Figure 5C).

#### 3.2.1 Thermoresponsive Materials

Gelatin is widely employed as a sacrificial material due to its excellent biocompatibility and reversible temperature‐dependent sol‐gel transition (Figure 5C) [175, 176]. Leveraging this property, Lewis and co‐workers used gelatin to create sacrificial vascular channels within a suspension matrix (Figure 5D) [159]. This approach enabled the fabrication of multiscale, perfusable vascular networks while preserving the viability and structure of the surrounding matrix. Similarly, Lee et al. reported the use of gelatin microspheres as a suspension matrix that, upon thermal liquefaction, generated micron‐scale pores resembling capillaries [41].

Although sacrificial molding strategy owns unique advantages, one of big issues it needs to consider is the residue of sacrificial materials. A novel approach by Sundaram et al. introduced gallium (Ga) as a sacrificial metal ink (Figure 5F) [161]. Ga is solid at room temperature but liquefies at 37°C, and exhibits an exceptionally strong capillary effect. This allows Ga to fully exit micron‐scale channels without residue, which is difficult to achieve with most polymeric materials. This strategy allowed the creation of high‐resolution vascular templates (20–500 µm) within collagen and fibrin matrices, maintaining excellent spatial fidelity and biocompatibility. Other thermoresponsive or dissolvable materials have also been investigated to broaden the range of fabrication flexibility [177]. Fang et al. utilized PF‐127 in combination with gelatin to expand the thermal control range for channel formation [175]. Carbohydrates are commonly found in food and medical applications, have also been used as sacrificial inks [178, 179]. Same group also demonstrated the successful 3D printing of carbohydrate‐based vascular templates, which were subsequently dissolved after matrix cross‐linking to create perfusable lumens [180].

#### 3.2.2 Chemically or Biologically Sensitive Materials

Sodium alginate is another widely used sacrificial material, known for its capacity to undergo ionic cross‐linking by divalent cations (e.g., Ca^2^^+^, Ba^2^^+^) and be selectively degraded by chelating agents (e.g., EDTA, sodium citrate) or alginate lyase (Figure 5C). This chemical responsiveness offers greater flexibility than purely thermoresponsive materials. For instance, Maiullari et al. [181] and Zhang et al. [182] used cross‐linked alginate scaffolds followed by EDTA‐mediated removal to generate vascular‐like cavities. Building on this approach, Lammers et al. [160] employed microfluidic spinning to fabricate alginate fibers, which were‐ then embedded within bulk hydrogels and enzymatically sacrificed (Figure 5E). The ECs in the matrix rapidly migrated into the resulting channels and interconnected, forming early‐stage vascular networks [183].

#### 3.2.3 Others

Beyond naturally derived hydrogels, synthetic polymers such as polyvinyl alcohol (PVA) have also shown promise as sacrificial materials. Zou et al. [184] utilized poly(vinyl alcohol) (PVA) as a sacrificial material to fabricate removable vascular templates, taking advantage of its water solubility and mechanically tunable properties. Together, these studies indicate that the effectiveness of sacrificial molding strategies depends less on fabrication complexity than on material‐specific properties, including phase transition behavior, cytocompatibility, removal kinetics, and compatibility with the host hydrogel.

### 3.3 Synthetic Polymeric Materials for Vascularized Cardiac Tissue Engineering

Beyond naturally derived biomaterials with inherent bioactivity, synthetic polymers have been extensively adopted in cardiac tissue engineering because their physicochemical properties can be independently and reproducibly controlled. Caspi et al. [103] developed porous polymeric sponges composed of equal fractions of poly(L‐lactic acid) (PLLA) and poly(lactic‐co‐glycolic acid) (PLGA). In this system, PLLA contributed mechanical stability, whereas the relatively faster degradation of PLGA created void spaces that promoted cellular infiltration and subsequent tissue remodeling.

Another widely used synthetic material in vascular tissue engineering is polycaprolactone (PCL). PCL is often processed into nanofibrous scaffolds via electrospinning techniques, which have been shown to support EC alignment and angiogenic activity [185]. Recently, conductive and elastic composite materials have attracted attention for MI therapy. A notable example is the construction of a prevascularized conductive elastic cardiac patch, in which a prefabricated poly (2‐hydroxyethyl methacrylate) (pHEMA) hydrogel was incorporated with holey graphene oxide and polypyrrole. Co‐culturing rat aortic ECs and CMs within this matrix, enabled the formation of functional vascular anastomoses and promoted strong electrical coupling with infarcted myocardium [186].

## 4 Methods for Engineering Vascularized Cardiac Tissues

Compared with 2D culture, 3D microtissues composed of different cell types can better simulate cell heterogeneity and organ function [187, 188]. Specially, cardiac tissue models are critical for studying cardiogenesis, cardiac diseases, and the evaluation of therapeutic strategies [3, 189, 190]. Currently reported vascularized cardiac tissues have various morphological styles, including pulsating cell spheroids [48], cardiac organoids with chamber‐like structure [10], myocardial tissue strips/rings [46], large‐scale cardiac patches [39], and perfusable organ‐on‐chips [40]. In the process of vascularized cardiac tissue constructing, different tissue engineering methods are involved, including cell aggregation [47], co‐culture [10], self‐assembly [11], mold‐casting [46], 3D bioprinting [48] and microfluidics [29, 40]. Different from the common definitions and classifications of cardiac organoids and engineered cardiac tissues, which have been systematically discussed in other reviews, we mainly focus on the different methods involved in constructing vascularized cardiac tissues, and classify different forms of vascularized cardiac tissues as follows.

### 4.1 Cardiac Microtissues by 3D Self‐Assembly

#### 4.1.1 Cardiac Spheroids by Multicellular Aggregation

**FIGURE 6:**
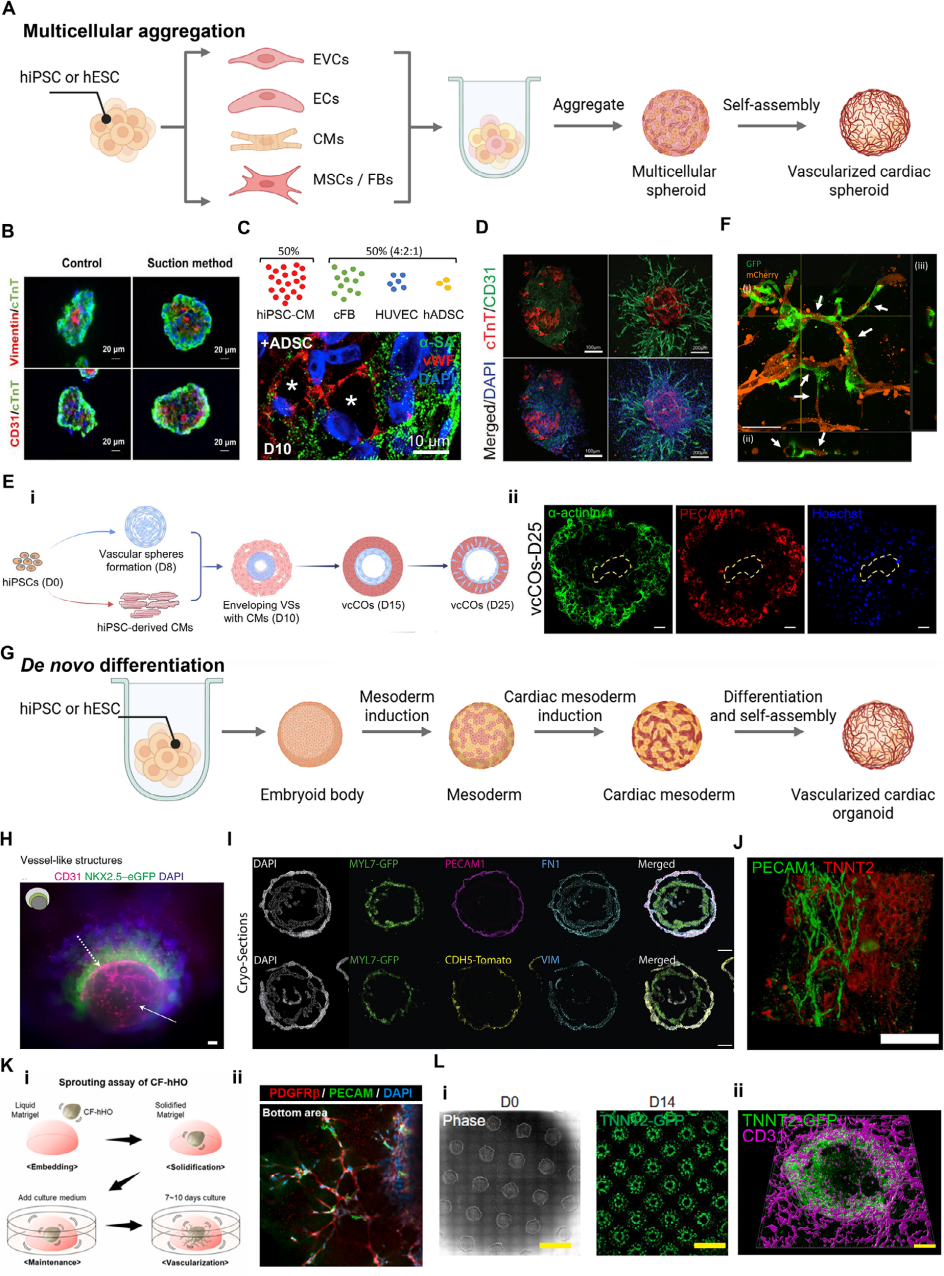
Vascularized self‐assembly cardiac spheroids and organoids via multicellular aggregation or *de novo* differentiation. (A) Schematic overview of self‐assembly fabrication of vascularized cardiac spheroids by multicellular aggregation. (B) Representative immunofluorescence images for cTnT (green), vimentin (red), and CD31 (red) in aggregated cardiac spheroids. Nuclei were stained with Hoechst 33342. Reproduced with permission [191]. Copyright 2023, Elsevier Inc. (C) Human adipose derived stem cells (hADSCs) improve vascularization and lumen‐like structure formation in human cardiac microtissues. Reproduced with permission [192]. Scale bar, 10 µm. Copyright 2017, Elsevier. (D) Immunofluorescence staining show CD31^+^ endothelial sprouts within vascularized cardiac spheroids. Scale bars, 200 µm. Reproduced with permission [48]. Copyright 2022, Wiley. (E‐i) Generation of chambered cardiac organoids with intrinsic vasculature; (ii) Whole‐mount immunostaining of vascular spheres (VSs) and vaschamcardioids (vcCOs‐cardiac organoids) for ECs (PECAM1, red) and CMs (α‐actinin, green). Scale bars, 50 µm [10]. Copyright 2024, Wiley. (F) Formation of hybrid vessels by culturing human cardiac spheroids in a microfluidic chip. Internal hiPSCs‐ECs (GFP) and external hiPSCs‐ECs (mCherry), demonstrate anastomotic connectivity. Scale bar, 100 µm. Reproduced with permission [29]. Copyright 2023, Elsevier. (G) Schematic overview of self‐assembly generation of vascularized cardiac organoids by *de novo* differentiation. (H) CD31 immunostaining reveals vessel‐like lumens formed by ECs in heart‐forming organoids. Scale bar, 100 µm. Reproduced with permission [193]. Copyright 2021, Springer Nature. (I) Cardioids demonstrate separation of CM and EC layers, with a vascular network layer surrounding the cardioid. Scale bars, 200 µm. Reproduced with permission [38]. Copyright 2021, Elsevier. (J) PECAM1 staining illustrates extensive branched vascular networks and tubular endothelial formations in self‐organized human heart organoid. Scale bar, 50 µm. Reproduced with permission [194]. Copyright 2021, Springer Nature. (K) Embedding hiPSCs‐derived heart organoid in Matrigel (i) results in formation of microvessel‐like structures confirmed by dual immunostaining for ECs (PECAM1) and pericytes (PDGFRβ) (ii). Reproduced with permission [95]. Copyright 2022, Elsevier. (L‐i) Micropatterned hPSCs under stage‐specific differentiation conditions results in spatially organized and vascularized cardiac organoid; (ii) 3D surface rendering shows vascular branches (CD31, magenta) in CMs (TNNT2, green). Scale bars, 4 mm, 4 mm and 200 µm, respectively. Reproduced with permission [11]. Copyright 2025, American Association for the Advancement of Science.

Compared with 2D cell co‐culture, 3D culture platforms more effectively recapitulate cell‐ECM interaction, tissue architecture, and nutrient/oxygen gradients, thereby enabling more physiologically relevant cell‐cell contacts and greater cell compaction, which more closely resemble the in vivo environment. 3D co‐culture of cardiac cells by cell aggregation provides a straightforward strategy to investigate cell interactions in cardiac spheroids or microtissues (Figure 6A). Toward that end, initial studies were conducted using primary CMs isolated from neonatal rat and cultured in hanging drops to allow spheroid formation, generating cardiac spheroids with an average volume of ∼ 1 × 10^7^ µm^3^ [47]. However, such animal‐derived spheroids fail to adequately reproduce human cardiac functions, limiting their utility for mechanistic studies and drug screening. With the advancement of hiPSCs culture and cardiac differentiation techniques, research focus has increasingly shifted toward the use of human‐derived cells.

Significant progress has been made in developing 3D cardiac models to recapitulate the structural and functional complexity of native myocardial tissue [14, 29, 48, 192, 195, 196, 197]. The cardiac spheroid diameter elevated from ∼ 200 to ∼ 300 µm upon the increasing initial number of seeded cells, ranging from 2500 cells to 10 000 cells, but only smaller microtissues showed a consistently higher spontaneous contraction rate [196]. With the development of hiPSCs‐derived CMs purification and large‐scale production of cell aggregates, more homogeneous cardiac spheroids with high sphericity were generated [191, 198] (Figure 6B). Meanwhile, native cardiac tissue is not solely composed of CMs, inspired by the contribution of supporting cell types such as cardiac FBs and vascular cells in developing heart, Richards et al. generated scaffold‐free functional cardiac spheroids with lumenized vascular network (Figure 6C) [192]. And they further developed MI model by incorporating these cardiac microtissues into an oxygen‐diffusion gradient, infarct cardiac organoids with a radius of ∼150 µm showed a higher intensity of hypoxia staining at the interior of live‐imaged organoids compared to organoids cultured in control conditions [28]. Subject to the limit of nutrient/oxygen diffusion, the dimensions of these cardiac spheroids were usually limited to about 200–300 µm in diameter [199]. Using co‐culture, cell aggregation and one‐step differentiation strategy with human EVCs and CMs, our previous report also generated vascularized cardiac microtissues with well‐organized microvasculature, and the development of microvasculature may help the cardiac microtissues to overcome size limitations (Figure 6D) [48]. Similarly, Yang et al. prepared hiPSCs‐differentiated vascular spheres, and then further wrapped the vascular spheres with hiPSCs‐derived CMs to form vascularized cardiac spheroids. Under a VEGF gradient, vascular cells migrated outward, effectively vascularizing the surrounding myocardium. This method successfully generated organoids with a high degree of vascularization, chamber structures, and complex cellular compositions (Figure 6E) [10]. These achievements highlight the potential of combining vascular and cardiac components to mimic native heart tissue in vitro, enabling functional integration of CMs with a vascular network. These vascularized cardiac models provide initial robust platforms for studying cardiogenesis, MI, and other cardiac diseases, while also serving as valuable tools for drug testing and regenerative medicine.

Giacomelli et al. demonstrated that 3D microtissues containing hiPSCs‐derived CMs, ECs, and cardiac FBs exhibited superior structural organization, contractile function, and metabolic maturation compared to those lacking one or more cell types. The synergistic presence of all three cell types was essential, underscoring the importance of intercellular crosstalk in myocardial development [14]. And by co‐culturing these hiPSCs‐derived, pre‐vascularized, cardiac microtissues with vascular cells in a microfluidic organ‐on‐chip, lumenized and interconnected vascular networks were formed through anastomosis, which were shown depending on continuous fluid flow perfusion. On the other hand, the presence of these microfluidic vascular networks allows aggregated cardiac spheroids to interconnect via perfusable vessels with an average length of 80 µm (Figure 6F) [29]. Moving forward, further optimization of cell ratios, incorporation of pericytes and SMCs, and integration of biomimetic biomaterials may enhance the structural and functional fidelity of these cardiac microtissues.

#### 4.1.2 Cardiac Organoids by *De Novo* Differentiation

Organoids are in vitro models that mimic organ formation and cellular heterogeneity, providing powerful platforms to study developmental processes, disease mechanisms, and therapeutic strategies [3]. Through the *de novo* progressive differentiation of hPSCs and multilineage development of human cardiovascular cells, cardiac organoids with diameters reaching the millimeter level have been successively constructed (Figure 6G) [3, 189]. hiPSCs‐derived cardiac organoids provide an experimental platform for reconstructing key stages of embryonic heart development, including early cardiogenesis [200]. Recent work has shown that self‐assembly‐based differentiation of hiPSCs can give rise to cardiac organoids containing vessel‐like structures, capturing early events of human cardiogenesis (Figure 6H) [193]. These studies support that the high self‐organizing capacity of cardiac mesoderm cells in generating mature cardiac organoids [201]. Building on this foundation, subsequent studies have reported the generation of chamber‐like cardiac organoids with diameters reaching the millimeter scale, in which CMs and ECs arise concurrently and organize into structures resembling native cardiac chambers (Figure 6I) [38]. Further structural analysis has yielded millimeter‐scale organoids with ventricular‐ or atrial‐like identities, where ECs line the inner luminal surfaces, indicating improved spatial patterning and tissue‐level organization [202, 203, 204].

Despite these advancements, the vascular networks in self‐assembled cardiac organoids remain relatively immature and often lack well‐defined, tube‐like vascular structures. To overcome this limitation, co‐differentiation of CMs and vascular cells has been induced under 3D culture conditions designed to more closely mimic the in vivo developmental microenvironment. Using a three‐step WNT modulation strategy, researchers generated cardiac organoids (∼1500 µm) that recapitulate the cellular composition, structure, and gene expression of the human fetal heart. These organoids feature internal chambers and organized multi‐lineage cardiac populations, reflecting key aspects of heart development. And a developed vascular network further supports their utility as advanced in vitro models (Figure 6J) [194]. Lee et al. demonstrated that embedding cardiac organoids (∼ 1500 µm in diameter) with ECs in Matrigel enabled the sprouting and extension of microvascular‐like networks with pericytes. Importantly, in vivo transplantation assays confirmed that these networks could connect to host blood vessels and maintain their functionality without external culture media. This study also underscored the critical role of the ECM in supporting the self‐assembly of well‐organized and high‐density vasculature (Figure 6K) [95]. Similarly, our previous work demonstrated that ECM is essential for the self‐assembly of well‐organized and high‐density microvasculature in co‐cultured EVCs and CMs [48]. A more recent study demonstrated that micropatterned hPSCs‐derived gastruloids enable in vitro modeling of the earliest stages of vascularization in cardiac organoids with spatially organized and branched vascular networks. These gastruloids further exhibit significant growth in the z‐axis (∼300 µm in diameter) and overall volume (1 × 10^8^ µm^3^). They identified a growth factor and small molecule cocktail that generated vascular networks in both cardiac and hepatic vascularized organoids, suggesting that a conserved developmental program is involved in creating the vasculature within different organ systems (Figure 6L) [11].

These observations indicate that effective reconstruction of the myocardial microenvironment requires coordinated incorporation of vascular and supporting cell populations. Interactions between CMs and non‐CM lineages, particularly vascular cells, contribute to tissue‐level organization, functional maturation, and the maintenance of homeostatic states [205]. Moving forward, further progress will likely depend on systematic optimization of cellular composition, including the relative proportions of cell types and the inclusion of additional populations such as immune and epicardial cells. Moreover, the exploration of paracrine signaling pathways and ECM components will provide deeper insights into the mechanisms driving cardiac tissue maturation and repair [206, 207].

### 4.2 Bioengineering Approaches for Vascularized Cardiac Constructs

#### 4.2.1 Engineered Vascularized Cardiac Tissues

**FIGURE 7:**
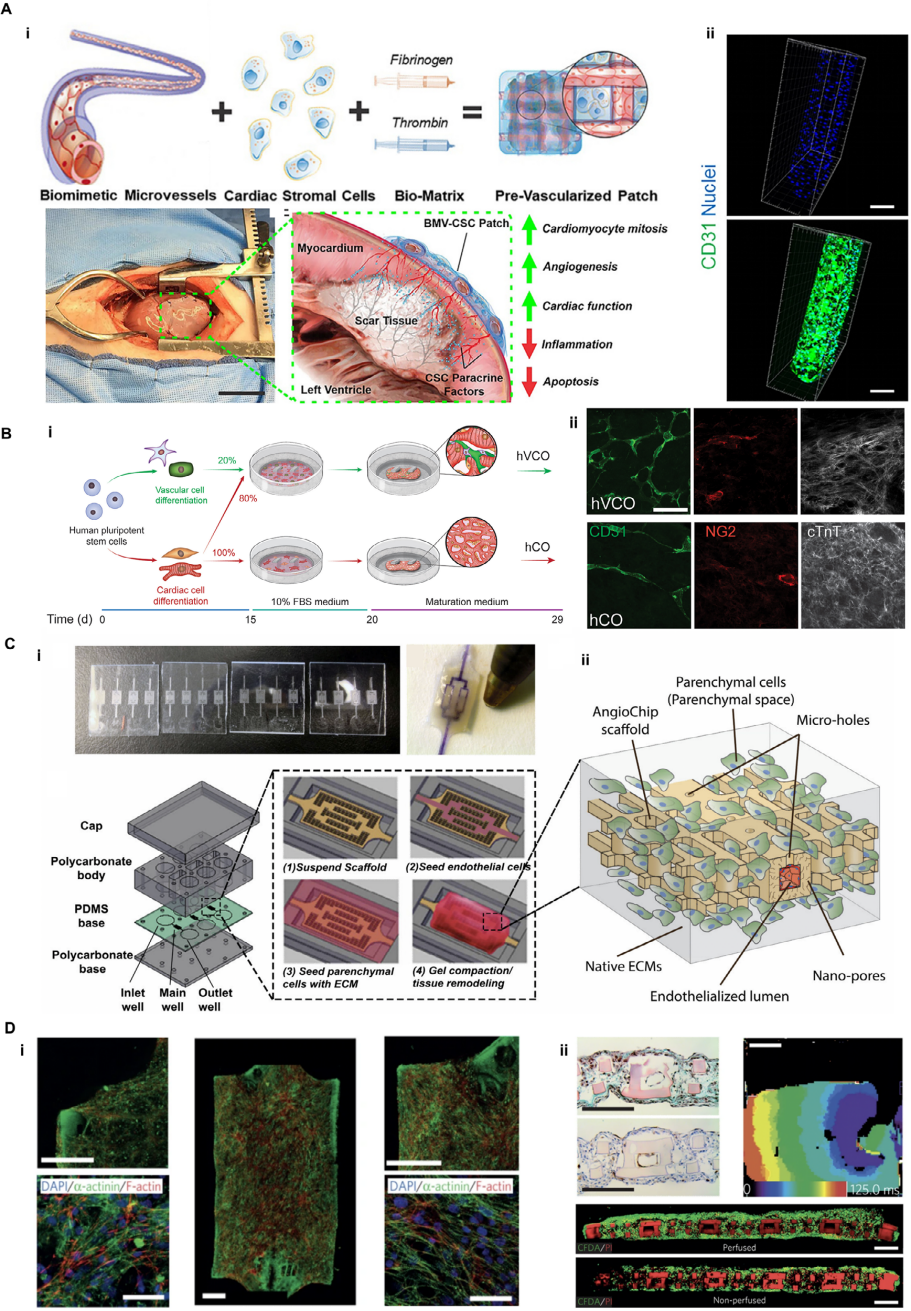
Casting and Microfluidic‐based strategies for engineering vascularized cardiac tissues. (A‐i) A schematic and intraoperative illustration of a prevascularized cardiac patch composed of biomimetic vessels, cardiac stromal cells and fibrin‐based bio‐matrix, which supports vascular integration and improves cardiac function via paracrine effects. Scale bar, 5 cm; (ii) Endothelialized biomimetic microvessels showed biomimetic vessel structure. Scale bars, 200 µm. Reproduced with permission [142]. Copyright 2020, American Chemical Society. (B‐i) Human vascularized cardiac tissue model generated via co‐culture of hPSCs‐induced vascular and cardiac lineages; (ii) Immunostaining for CD31, NG2, and cTnT reveals enhanced vascular and myocardial structure in vascularized cardiac model compared to conventional one. Scale bars, 50 µm. Reproduced with permission [46]. Copyright 2023, Cell Press. (C‐i) Stepwise fabrication of the AngioChip platform integrates endothelialized lumens, and perfusable vasculature to support in vitro tissue remodeling; (ii) Exploded‐view schematic and structural diagram show scaffold architecture and cellular spatial distribution. Reproduced with permission [209]. Copyright 2016, Springer Nature. (D‐i) Immunostaining and functional characterization of the engineered cardiac tissues. Scale bars, 50 µm (left and right), 500 µm (middle); (ii) Hematoxylin‐eosin staining and optical mapping confirm myocardial tissue integration and electrical conductivity, while fluorescent perfusion assays (CFDA/VI) indicate functional vascular perfusion under flow versus static conditions. Scale bars, 1 mm (top three image), 200 µm (bottom two image). Reproduced with permission [209]. Copyright 2016, Springer Nature.

Vascularization of engineered heart tissues (EHTs) involves the coordinated interplay of cellular composition, scaffold design, culture dynamics, and biofabrication strategies. One approach has focused on incorporating functional features directly into scaffold architectures to guide vascular organization. In addition, functional composite materials such as hGO/PyP [142, 186] or microvessels have been introduced into cardiac patches to improve mechanical performance, facilitate cell attachment, and enhance vascular ingrowth, ultimately contributing to MI repair (Figure 7A). Beyond material‐based strategies, cellular interactions also play a crucial role in driving cardiac tissue maturation and vascular development [46, 208]. To dissect the mechanisms by which vascular cells influence myocardial maturation, Voges et al. generated engineered cardiac organoids comprising vascular ECs, epicardial cells, FBs, and CMs that self‐organized into in vivo‐like EHT constructs. Mechanistic analysis revealed that paracrine signals derived from vascular cells, particularly PDGFB and LAMA5, enhanced CM function and promoted the expression of maturation‐associated markers (Figure 7B) [46].

Given that MI results in irreversible CM loss and poor regenerative capacity, the development of centimeter‐scale vascularized cardiac patches has been pursued to replenish lost myocardium. One widely adopted fabrication strategy is the mold‐casting approach, which enables spatial control over tissue architecture and cellular composition [210]. Optimizing cellular composition (CMs, ECs, and stromal cells) improves vascularization and electromechanical integration. Notably, in vivo pre‐vascularization strategies, such as omentum or renal capsule embedding, can further enhanced these outcomes [211]. These pre‐vascularized patches demonstrated improved structural and electrical integration with host myocardium and mitigated adverse ventricular remodeling post‐MI. However, the casting strategy for tissue preparation presents several inherent limitations. For instance, Sundaram et al. employed gallium as a sacrificial material to create capillaries within the matrix [161]. Although this perfusion‐based endothelial seeding approach enabled initial vascularization, it often resulted in non‐uniform cell distribution and poor cell adhesion under high shear stress. Ensuring cell viability and maintaining barrier integrity in capillary‐scale vessels remain significant challenges. Additionally, casting techniques frequently suffer from restricted CM infiltration, heterogeneous endothelial coverage, and mass transport limitations. These limitations highlight the need for advanced biofabrication strategies that integrate biological self‐organization with precise structural control.

#### 4.2.2 Engineering Perfusable Vascularized Cardiac Tissues

To enhance vascularization under controlled conditions, some researchers have utilized bioreactor systems [212]. These systems provide dynamic culture conditions, such as continuous medium perfusion, which significantly improve vascular and cardiac tissue development [213]. Studies have shown that perfusion‐based culture facilitates the formation of densely packed proto‐tissue composed of vascular‐like and cardiac‐like cells. Upon in vivo implantation, bioreactor approach allows for the precise control of nutrient and oxygen gradients, which are essential for the development of organized vascular networks and tissue maturation [168]. Building on these insights, numerous efforts have been made to fabricate vessel‐mimicking cardiac tissues using mold‐based casting methods. These templates can be either designed artificially [163] or obtained directly from tissues via decellularization strategies [214].

An emerging area of research focuses on microfluidic organ‐on‐a‐chip models, which provide anatomically and physiologically relevant platforms for studying cardiac tissue development and vascularization. These heart‐on‐a‐chip systems allow for controlled nutrient delivery, dynamic culture conditions, and improved vascular functionality. Despite these advancements, replicating hierarchically organized, multi‐level vascular networks capable of sustaining long‐term function, particularly in larger engineered tissues, remains a major challenge. By addressing these challenges, Milica Radisic and co‐workers advanced the microchip technology, called AngioChip, a scaffold that supports the assembly of parenchymal cells on a mechanically tunable matrix surrounding a 3D, perfusable, branched microchannel network coated with ECs (Figure 7C). When CMs were added to this scaffold, millimeter‐thick (1.75–2 mm thick) cardiac tissues with integrated vasculature were successfully constructed. The channels are intricately engineered to be lined with a continuous monolayer of ECs, faithfully replicating the inner lining of blood vessels. Surrounding these channels, the external compartment is populated with parenchymal cells relevant to the target tissue or organ. Importantly, the channel walls are designed with a hierarchical porous structure, incorporating both microscale apertures and nanoscale pores. This dual‐porosity architecture facilitates the bidirectional diffusion of soluble factors, nutrients, waste products, and therapeutic agents between the vascular lumen and the surrounding parenchymal space, thereby more accurately recapitulating in vivo physiological microenvironments. This design supports vascular perfusion and facilitates integration with host vasculature following implantation. The close spatial and biochemical coupling between endothelial and cardiac cells within this system enables physiologically relevant electromechanical interactions, influencing CM contractility, conduction, and survival (Figure 7D). As a result, such integrated platforms provide a robust in vitro framework for investigating ischemia‐reperfusion injury, myocardial inflammation, and drug‐induced arrhythmias in a human‐relevant context [209, 210, 211, 212, 213, 214, 215, 216].

#### 4.2.3 Other Bioengineering Approaches for Cardiac Vascularization

Nature provides additional inspiration for vascular network design, particularly through the intricate architecture of leaf vein systems, which exhibit remarkable structural and functional similarities to mammalian vasculature. Drawing on this natural blueprint, the leaf‐venation‐directed (LVD) approach used the geometrical patterns of leaf veins as topological templates for vascular network fabrication. By casting fibrin hydrogels onto leaf vein templates, researchers have created aligned, cell‐laden constructs with hierarchical microchannels, offering a cost‐effective strategy to build perfusable vascular networks in thick engineered tissues [217]. LVD‐based platforms offer a promising strategy to overcome vascular complexity in cardiac tissue engineering and enable scalable, physiologically relevant constructs.

### 4.3 Fabrication of Vascularized Cardiac Tissues with 3D Bioprinting

3D bioprinting has become an important tool for cardiac tissue fabrication, including the ability to create patient‐specific models using the patient's own cells, reducing the risk of immune rejection [218, 219]. Beyond customization, bioprinting enables spatial control over the distribution of cells and biomaterials, replicating the complex architecture of heart tissue and integrating vascular structures for improved oxygen and nutrient delivery.

#### 4.3.1 Direct Printing

**FIGURE 8:**
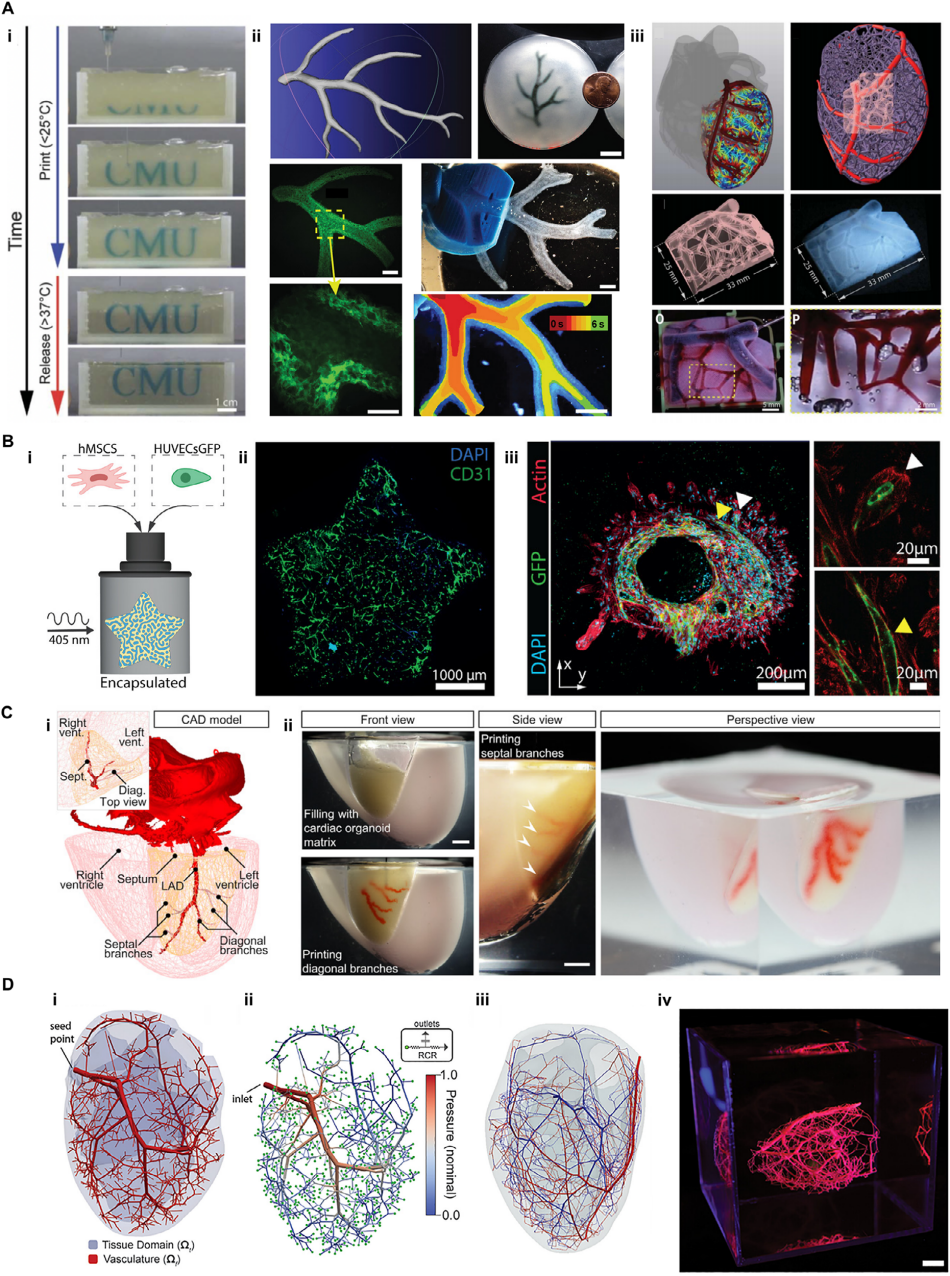
3D bioprinting strategies for engineering vascularized cardiac tissues. (A‐i) FRESH printing of the letters “CMU” in the gelatin support, and unapparent deformation of the letters during supporting materials melting is observed. Scale bar, 1 cm; (ii) A model of a section of a human right coronary arterial tree from 3D MRI is printed by FRESH method. Scale bars, 10, 2.5, 2.5, 1, and 2.5 mm (left to right and top to bottom); (iii) Perfusion imaging and computational flow mapping indicating lumen patency and flow dynamics. Anatomically relevant vascularized cardiac constructs featuring multiscale, interconnected networks suitable for volumetric myocardial tissue engineering. Scale bars, 5 mm (left) and 2 mm (right). Reproduced with permission [227]. Copyright 2015, American Association for the Advancement of Science. (B‐i) Schematic showing the volumetic bioprinting of phase‐separating gels with cells suspended in; (ii) Confocal tile scan of cell‐laden bioprinted star. Scale bar, 1000 µm. (iii) Confocal tile scan of cell‐laden bioprinted perfusable construct. Scale bars, 200 µm (left), 20 µm (right). Reproduced with permission [228]. Copyright 2026, Wiley. (C‐i) CAD‐based vascular patterning and (ii) front, top, and volumetric views of printed cardiac models containing perfusable septal and diagonal branches. Scale bars, 5 mm. Reproduced with permission [159]. Copyright 2019, American Association for the Advancement of Science. (D‐i) Open‐loop synthetic vasculature tree with 1000 outlets; (ii) Matched CFD simulation generated from the 1000‐outlet tree; (iii) Connections between synthetic arterial and venous trees form a closed‐loop network; (iv) Orthogonal view of printed biventricular. Scale bars, 1 cm. Reproduced with permission [229]. Copyright 2025, American Association for the Advancement of Science.

Advances in digital light processing (DLP)‐based and extrusion‐based bioprinting have enabled precise spatial patterning of cells and biomaterials, facilitating the fabrication of vascularized cardiac tissues [220, 221, 222]. Extending these strategies, cardiac tissues with microvasculature were achieved via 3D bioprinting and one‐step differentiation method [48]. Related approaches combining 3D printing with one‐step differentiation have also produced cardiac tissues containing microvascular features. However, one of the persistent challenges lies in the mechanical weakness of many hydrogel‐based bioinks, which limits resolution and structural fidelity [223, 224]. To address this, researchers have introduced supportive materials and novel printing strategies. For example, Dvir et al. [56] developed a suspension printing process using alginate‐based media, enabling the fabrication of heart‐like structures with intricate internal cavities (Figure 8A). In another study, Annabi et al. [225] utilized dual‐nozzle extrusion with MeTro/GelMA bioinks to spatially organize CMs, ECs, and FBs, producing vascularized cardiac tissues with large lumens. The elastic properties of MeTro closely approximate native myocardial mechanic, making it particularly well‐suited for cardiac tissue engineering. In addition to biophysical factors, ECM cues, angiogenic signals, and stem cell‐derived modulators have been increasingly recognized for their roles in guiding vascular and myocardial development [226]. Incorporating these biological signals into bioprinted constructs may further enhance vascular integration and tissue functionality.

Since the advent of additive manufacturing four decades ago, 3D printing has remained anchored to layer‐by‐layer fabrication, depositing material as discrete voxels, lines, or planes that are stacked into a 3D object [230]. Recently, volumetric 3D printing has emerged as an alternative technology that overturns this paradigm by enabling the fabrication of centimeter‐scale constructs within seconds [231, 232, 233]. Volumetric printing is defined as applying a controlled energy field, such as light [231, 233, 234, 235, 236, 237, 238, 239, 240, 241], sound [242, 243], or similar to a photosensitive resin or analogous material all at once within a 3D volume, thereby inducing selective solidification or phase transitions simultaneously throughout the bulk [244]. When applied to cell‐laden structures, volumetric printing demonstrates excellent cell viability and has already been employed in numerous in vitro tissue constructs. For instance, Lian et al. employed volumetric printing to successfully fabricate a cardiac structure using CM‐laden decellularized ECM within 45 s, and observed synchronized contractions of CMs on day 10 (Figure 8B). [245]. Other studies have demonstrated high‐precision printing of tissue models with complex vascular networks, further confirming the excellent biocompatibility of this approach [241, 246, 247]. Although volumetric bioprinting has not yet been directly applied to vascularized cardiac tissue, these results suggest its potential utility for advancing cardiac tissue engineering.

#### 4.3.2 Sacrificial Printing

Sacrificial 3D bioprinting creates perfusable lumen networks by removing a temporary printed template, which can subsequently be endothelialized to form vascular structures [56, 180]. Materials that have been used for sacrificial printing to fabricate vascularized cardiac tissues include carbohydrates [196], PF‐127 [248], gelatin [45], and others.

Building on this technology, Lewis and colleagues further advanced the sacrificial printing strategy by writing a viscoelastic fugitive ink into acellular hydrogels and silicone matrices (3D Bioprinting of Vascularized, Heterogeneous Cell‐Laden Tissue Constructs) [159] (Figure 8C). After curing, the sacrificial ink was removed, leaving behind a 3D network of interconnected channels. Building upon this progress, the same group developed the sacrificial writing into functional tissue (SWIFT) technique, wherein gelatin‐based sacrificial bioink is extruded into clusters of hiPSCs‐derived CMs. After cross‐linking the cellular matrix, the sacrificial ink is dissolved, creating hollow perfusable channels that are subsequently seeded with ECs to form mature vascular structures. Inspired by the SWIFT framework, Xiong et al. introduced a modified approach by replacing cell aggregates with GelMA microparticles to construct perfusable ventricular tissues featuring freeform vascular networks [175].

Recently, a new approach introduced ESCAPE (Engineered Sacrificial Capillary Pumps for Evacuation) molding, a novel fabrication method that leverages gallium‐based sacrificial templates to construct multi‐level vascularized architectures within various hydrogels [161]. Unlike conventional additive biomanufacturing, which is constrained by nozzle size and resolution limits, ESCAPE enables geometry construction by leveraging gallium's unique phase properties to achieve sub‐10 µm precision. This approach enables the reproducible formation of continuous, perfusable vascular trees spanning from arteriolar (∼300 µm) down to microvascular (<10 µm) scales. As a result, ESCAPE molding avoids nozzle‐based resolution limits and supports the construction of vascular spanning arteriolar to capillary scales. The approach also allows patterning of luminal topography, providing a means to guide endothelial alignment and model disease‐relevant vascular features.

Despite advances in 3D bioprinting techniques, one critical step remains underexplored: the rational design of physiologically relevant, tree‐like vascular architectures. Most engineered tissues lack direct anatomical templates for vasculature, leading to simplistic lattice‐based perfusion schemes that inadequately support cell survival with high densities. While various algorithms have attempted to generate vascular networks, they often suffer from limited scalability and computational inefficiency [249]. To address this, Sexton et al. recently introduced a model‐driven design platform that integrates hemodynamic parameters, such as flow rate, pressure, and branching hierarchy, to generate complex, perfusable vascular trees. This platform enables rapid design of organ‐specific vasculature and demonstrated significantly improved cell viability in 3D bioprinted constructs [229] (Figure 8D). Compared with previous approaches, this platform not only accelerates vascular network generation but also incorporates biophysical constraints to improve physiological fidelity. Together, these developments illustrate how sacrificial bioprinting strategies can be combined with design‐based approaches to advance the fabrication of vascularized cardiac tissues.

#### 4.3.3 4D Bioprinting

Beyond conventional 3D printing strategies for fabricating vascularized cardiac tissues, time has emerged as an additional design dimension, giving rise to the rapidly evolving field of 4D bioprinting. In this approach, printed constructs are engineered to dynamically alter their shape, structure, or function over time in response to external stimuli, such as temperature [250, 251], pH, light, or magnetic fields, or internal biological cues, including cell differentiation and cell‐generated forces [252, 253]. For example, exploiting the intrinsic shrinkage of PNIPAM at critical solution temperature (above 32°C), Sapir‐Baruch et al. [254] engineered nanoscale PNIPAM particles whose volume can be controllably and reversibly triggered, and embedded them within an ECM‐based hydrogel to create a hybrid bio‐ink for printing vessels within myocardial tissue. The printed vascular structures underwent post‐fabrication constriction to reach capillary‐scale dimensions, thereby overcoming the resolution constraints to current printing methods.

Leveraging the swelling characteristics of GelMA, Cui et al. fabricated a mechanically adaptive cardiac patch capable of 4D shape transformation [255]. Following implantation, the construct adjusted dynamically to the cyclic motion of the beating heart, establishing mechanical coupling with the surrounding myocardium. This adaptive interaction provided biomechanical cues that promoted vascular network formation and CM maturation in vitro. Importantly, these effects were preserved after implantation, resulting in improved cell retention and enhanced vascular perfusion in a murine model of chronic MI.

## 5 Vascularized Cardiac Tissues In‐a‐Dish: Potential Diseases to Model

The maturation of hiPSCs technologies has enabled the systematic use of large hiPSCs cohorts for drug screening and disease modeling. This has facilitated the development of hiPSCs‐based cardiovascular disease models, particularly for monogenic disorders with well‐characterized genotype‐phenotype correlations [256]. Alongside genetic modeling efforts, advances in biofabrication and biomanufacturing have shifted in vitro cardiac models from simple 2D monolayers into complex 3D organoids and EHTs. These platforms more closely reproduce key structural and functional characteristics of native myocardium and have therefore expanded the scope of cardiac disease modeling, as summarized in Table 1.

**TABLE 1:** Summary of approaches to cardiomyocyte‐associated cardiovascular disease modeling.

Diseases	Phenotype	Cell type		Model method
long QT syndrome (LQTS)	Prolonged QT interval and abnormal T wave are prone to malignant arrhythmias, leading to recurrent syncope, cardiac arrest and even sudden death and other cardiac adverse events	hiPSCs‐CMs	Successful differentiation to CMs, using iPSCs derived from patients with type 2 LQTS (A614V missense mutation in the KCNH2 gene) in which heterozygous mutations of A614V were found	[2D] [257]
			Patient‐specific type 3 LQTS‐iPSCs derived myocardium filled with synthetic filamentous matrix to develop a 3D in vitro model that exhibited varying degrees of contractile abnormalities	[Bioprinting technologies] [258]
			EHT was constructed using hiPSCs‐CMs, and it was successfully induced to exhibit LQTS characteristics with drugs	[EHT] [259]
		hiPSCs‐CMs + hiPSCs‐ECs + hiPSCs‐FBs	Type 1 LQTS hiPSCs differentiate into CMs, ECs, and FBs. The three cell types are combined to form a 3D spheroid that exhibits a longer field potential duration	[Cardiac spheroids] [260]
		Type 2 LQTS‐CMs + FBs	hiPSCs‐CMs and FBs were co‐cultured with porcine dECM in microfluidic chips, and it was found that the presence of LQTS in both types of cells was more obvious	[Organ‐on‐a‐chip] [8]
MI/ischemic heart disease	The overall progression from myocardial injury to complete remodeling can be divided into three stages: stressed CM death, inflammatory response, and fibrotic scarring. Death, inflammation, and fibrotic scarring, ultimately impairing myocardial contractility and electrophysiology	Primary CMs + FBs	FBs activated by TGF‐β affect CM monolayer conduction velocity	[2D] [261]
		Primary CMs + ECs + FBs	Cardiac spheroids were formed using three types of cells and treated with TGF‐β1, and a significant increase in collagen deposition was observed. Treatment with doxorubicin reveals apoptosis and disruption of the vascular network	[Cardiac spheroids] [262, 263]
		hiPSCs‐CMs	The hypoxia culture was used to simulate ischemic heart disease, and the reoxygenation was used to simulate reperfusion, and the disintegration of sarcomere was observed	[2D] [263]
			Cardiac organoids using an oxygen diffusion gradient and stimulated with the neurotransmitter norepinephrine mimic the structure of the human heart after MI	[Cardiac organoids] [28]
		hiPSCs‐CMs + hiPSCs‐FBs	hiPSCs‐CMs and hiPSCs‐FBs were printed in dhECM mixed hydrogels with GelMA and MeHA	[Bioprinting technologies] [28, 264]
			TGF‐β1 stimulation to recapacitate the microenvironment in which fibrosis occurs in MI	
		hESCs‐CMs	Implantation of hESCs‐CMs can effectively restore the function of the MI area	[2D] [265]
Diabetic cardiopathy	Cardiac enlargement, fibrosis, and dysfunction	hESCs‐CMs	Based on hESCs‐CMs, modulate the WNT signaling pathway to form cardiac organoids and mimic diabetic cardiomyopathy	[Cardiac organoids] [194]
			The use of hESCs‐CMs to construct EHT, and the use of advanced glycation end products to induce the expression of diabetic cardiomyopathy characteristics	[EHT] [266]
Hypertrophic cardiomyopathy	It is characterized by left ventricular thickening and diastolic dysfunction, myocardial hypertrophy and derangement, and increased myocardial fibrosis, diastolic dysfunction	hiPSCs‐CMs + ECs + FBs	Micro‐pattern hiPSCs‐CMs from hypertrophic cardiomyopathy patients showing impaired diastolic function, prolonged relaxation time, decreased relaxation rate, and shortened diastolic sarcomere length as evidenced	[2D] [267]
			Organoids were formed using patient‐derived hiPSCs‐CMs, ECs, FBs from cardiomyopathy patients to mimic hypertrophic cardiomyopathy	[Cardiac organoid] [268, 269]
			Up to 8 months of electroregulation induces hiPSCs‐CMs‐constructed cardiac organoids to model polygenic left ventricular hypertrophy	[EHT] [270]
Dilated cardiomyopathy	Most patients have enlarged or dilated left ventricles and systolic dysfunction	hiPSCs‐CMs	hiPSCs were derived from the patient and further differentiated into CMs	[2D] [271]
			Cardiac spheres were formed with the help of patient‐derived iPSCs, and the role of peptidyl arginine deiminase was studied	[Cardiac spheroids] [272]
			Use TTN to truncate variations iPSCs builds EHT	[EHT] [273]
Heart failure	Interstitial fibrosis results in reduced cardiac remodeling and ventricular compliance.	hiPSCs‐CMs	Activation of TGF‐β signaling	[EHT] [264]
			Adjusting the ratio of FBs	[Organ‐on‐a‐chip] [274], [Cardiac spheroids] [275]
		Primary CMs + FBs	Cyclic mechanical compression	[Cardiac spheroids] [276], [bioprinting] [277]

However, the absence of functional vasculature has long constrained the physiological relevance of early cardiac tissue models. Many systems lacked perfusable vascular networks, limiting oxygen and nutrient transport and reducing their utility in drug testing [278]. To address this limitation, recent studies have incorporated vascular cell populations and perfusion‐enabled culture systems, leading to improved tissue viability and more faithful reproduction of disease‐relevant phenotypes. Consequently, vascularized cardiac platforms, including spheroids, organoids, EHTs, and 3D‐printed constructs, are increasingly being explored for disease modeling and drug toxicity screening (Table 2). These systems are particularly well suited for investigating vascular‐associated cardiac pathologies, including EC‐, SMC‐, MSC‐, and FB‐related diseases. The presence of a functional vascular compartment supports sustained tissue survival and enables modeling of key pathophysiological features, such as hypoxia, inflammation, fibrosis, and endothelial dysfunction. When integrated with MPS, which simulate dynamic fluid flow, mechanical loading, and biochemical gradients, these vascularized models can more accurately mimic native hemodynamic conditions and predict therapeutic responses. Such integrated platforms provide an experimental framework for studying the progression of vascular‐centric cardiac diseases and evaluating interventions targeting angiogenesis, vasodilation, anti‐thrombotic responses, and endothelial repair.

**TABLE 2:** Summary of approaches to non‐cardiomyocyte‐associated cardiovascular disease modeling.

Cell type	Disease	Pathogenesis	Phenotype		Model method
hiPSCs‐ECs	LMNA vascular injury	LMNA mutations	Left ventricular enlargement or reduced systolic function is frequently associated with conduction system disease. Clinical endothelial dysfunction, impaired angiogenesis and nitric oxide (NO) production	hiPSCs‐ECs models were derived from patients with LMNA‐associated dilated cardiomyopathy who exhibited decreased function of hiPSCs‐ECs	[2D] [279, 280]
	Hypoplastic left heart Syndrome	Genetic factor	Complex congenital heart disease characterized by abnormalities in the left ventricle, associated valves, and ascending aorta	A developmentally impaired endocardial population was identified in HLHS. Subsequent functional assesses using purified iPSC‐derived ECs showed that endocardial defects in HLHS can lead to endocardial to mesenchymal transformation and impaired angiogenesis	[2D] [281]
	Alcohol‐associated coronary artery disease risk	Genetic and environmental factors	The main blood vessels that supply blood to the heart (coronary arteries) struggle to deliver enough blood, oxygen and nutrients to the heart muscle Coronary EC dysfunction	Using iPSCs‐ECs and CRISPR/Cas9‐corrected ALDH2*2 iPSCs‐ECs, author modeled ALDH2*2‐induced EC dysfunction in vitro, demonstrating an increase in oxidative stress and inflammatory markers and a decrease in NO production and tube formation capacity	[2D] [282]
	Familial PAH	BMPR2 mutations	Increased pulmonary vascular resistance and pulmonary artery pressure	iPSCs‐ECs could provide a unique platform to model the propensity to FPAH ‘in‐a‐dish’	[2D] [283]
				iPSCs‐ECs had similar functional abnormalities and altered gene expression patterns to pulmonary artery ECs from patients	[2D] [284]
	Early‐onset atherosclerosis	Repeated, minor damage (stress, inflammation, Chemical substance) to the lining of the artery (endothelium)	Plaques capable of invading the lumen of the middle and great arteries (containing lipids, inflammatory cells, smooth muscle cells, and connective tissue)	Treating hiPSCs‐ECs with FFA or oxLDL. Functional changes: high ROS production, reduced NO synthesis were observed in the hiPSCs‐ECs vessels‐on‐chip	[Organ‐on‐a‐chip] [285]
	Arrhythmogenic cardiomyopathy (ACM)	Mutations in desmosomal genes	Arrhythmias and fibro‐fatty replacement of the myocardium	Using ACM hiPSCs from a patient carrying the heterozygous c.2013delC PKP2 mutation, and generating CTRL‐ and ACM‐MTs by combining CTRL hiPSCs‐CMs and ECs with either CTRL hiPSCs‐cardiac FBs or skin FBs or with ACM hiPSCs‐cardiac FBs or skin FBs	[EHT] [14]
	Long‐COVID‐associated cardiac dysfunction	EC‐released cytokines contribute to cardiac dysfunction	Cardiac dysfunction	Cardiac organoids comprising iPSCs‐ECs and iPSCs‐CMs showed cardiac dysfunction after severe acute (COVID) exposure	[Cardiac organoid] [286]
hiPSCs‐SMCs	Coronary artery disease susceptibility	Genetic and environmental factors	Increased pulmonary vascular resistance and pulmonary artery pressure	iPSCs generate vascular smooth muscle cells (VSMCs). Risk VSMCs exhibit globally altered transcriptional networks that intersect with previously identified coronary artery disease risk genes and pathways, concomitant with aberrant adhesion, contraction, and proliferation	[2D] [287]
				SMC‐specific deletion of Smad3 in a murine atherosclerosis model resulted in greater plaque burden, more outward remodelling and increased vascular calcification	[2D] [288]
MSCs	Torsade de Pointes	Lethal arrhythmia that is often drug‐induced	Polymorphic EFP and meandering spiral wave re‐entry	Treating with IKr channel blockers	[Cardiac tissue sheets] [289]
FBs	Fibrosis‐induced heart failure	Cardiac and systemic factors	High collagen deposition, increased tissue stiffness, BNP secretion, and passive tension. Force of contraction was significantly decreased	Co‐cultured human cardiac FBs and iPSCs‐CMs in microfabricated device with live force measurement capabilities. TGF‐β was used as a trigger for fibrosis	[Organ‐on‐a‐chip] [274]
				hiPSCs‐CMs are cocultured with either 25% or 75% ventricular cardiac FBs to model normal and fibrotic myocardium	[Organ‐on‐a‐chip] [290]
				3D cardiac sphere platform composed of hESCs‐CMs and MSCs, TGF‐β1 is a primary inducer of cardiac fibrosis	[Cardiac spheroids] [291]

## 6 Limitations and Prospects

To examine strategies for establishing perfusable vasculature in engineered cardiac tissues, we performed a comparative analysis of fabrication components and bioengineering methodologies used to generate vascularized myocardial constructs. Rather than relying on a single technique, these approaches differ in how they influence cellular composition, vascular organization, and cell‐matrix interactions (Table 3). Among current strategies, 3D self‐assembly‐based systems permit high cell density and the incorporation of multiple cardiac cell populations under material‐minimal conditions, which has been associated with improved CM maturation. However, the use of hiPSCs‐derived CMs, the primary cellular source for cardiac tissue assembly, remains hindered by several biological challenges, including inter‐line variability, scalability, and an immature phenotype characterized by disorganized sarcomeric structures, limited calcium handling, and electrophysiological instability. In addition, differentiation protocols frequently yield heterogeneous mixtures of atrial‐, ventricular‐, and nodal‐like subtypes, increasing arrhythmogenic risk and complicating synchronized tissue function [201, 202, 292, 293].

**TABLE 3:** Summary of strategies for vascularization.

Strategies		Advantages	Limitations	Applicable scales	References
3D self‐assembly	multicellular aggregation	Material free—Physiological cell density Rapid self‐assembly of micro‐vessels Authentic cell–matrix crosstalk Modular tessellation Fast anastomosis in vivo Low immunogenicity & drug accessibility	Poor geometrical programmability Diffusion‐limited thickness High batch‐to‐batch variability Low mechanical strength Random electrical orientation Scale‐up bottleneck	Capillary (< 20 µm)	[47, 192, 197]
	de novo differentiation	Material free—Physiological cell density Built‐in cell pairing Enhanced CM maturation HTS‐friendly disease models	Difficult synchronisation of differentiation windows Limited fine control of final cell ratio Conflicting metabolic demands Vessel calibre restricted to capillaries Poor long‐term stability	Capillary (< 20 µm)	[38, 194]
Molding and casting	/	Controllable geometry and channels Low cost & simple protocol Immediate perfusion capability Pre‐engineered anisotropy Easy surface functionalisation	Template‐removal damage risk Complex fabrication of small‐diameter & highly branched networks Limited material choices Channel closure/collapse risk Lack of native micro‐vascular plasticity	20 µm – 25mm	[46, 142, 294]
Microfluidics	/	Micron‐scale channels match physiological dimensions Instant perfusion & controllable micro‐environment Precise co‐culture and mechanical stimulation High‐throughput drug/toxicity screening Seamless integration with sensors	Basically “2D layer‐stacking” fabrication Cannot produce suture‐ready large vessels in one step Limited seeding density Long fabrication time & high cost Poor long‐term culture stability	20 – 300 µm	[142, 163, 209]
3D bioprinting	Direct printing	One‐step fabrication & streamlined workflow High architectural programmability Instant perfusion & rapid anastomosis Multi‐material / multi‐cell co‐deposition per layer Seamless integration with imaging data High throughput & clinically scalable	Resolution vs. diameter trade‐off Shear stress & phototoxicity Thickness–perfusion bottleneck Narrow material window Lack of long‐term vascular remodelling Cost and regulatory barriers	250 µm – 4mm	[181, 182]
	Sacrificial printing	Freely designed channel size and trajectory Decoupling of ink and bulk‐matrix choice One‐step co‐localisation of “muscle + vessels” Instant perfusion and rapid host anastomosis Wide material repertoire Compatible with multi‐material printing	Additional sacrificial‐removal step complicates workflow Small‐diameter channels prone to collapse/breakage Residual toxicity of sacrificial ink Removal efficiency drops at thickness >2 mm Lack of native micro‐vascular plasticity Weak interfacial bonding	20 µm – 5 mm	[159, 175]
	4D bioprinting	“Post‐shrink” boosts effective resolution Programmable hierarchical stiffness & aligned micro‐structure Compatible with multi‐cell co‐printing	Stimulus conditions can be cytotoxic Shape‐memory cycling stability is limited Narrow material window Lack of long‐term vascular remodelling Thickness–deformation uniformity challenge	20 µm – 3 cm	[228]
Mix	in vivo transplantation + molding and casting	Rapid vascularization Higher vascular maturity	Interspecific differences Uncertainty in in vivo survival and functional differentiation of seed cells Risk of in vivo immune rejection and inflammatory response	20 µm – 25mm	[211]
	Sacrificial printing + molding and casting	Customizable multi‐level bionic vascular channels Wide selection range of scaffold materials	Template removal process risks damaging scaffold structure The interface compatibility between channels and cells needs to be optimized	20 µm – 5 mm	[180]
	Sacrificial printing + in vivo transplantation	Rapid vascularization Higher vascular maturity Customizable multi‐level bionic vascular channels	Interspecific differences Uncertainty in in vivo survival and functional differentiation of seed cells Risk of in vivo immune rejection and inflammatory response	20 µm – 5 mm	[56]

Techniques such as molding and casting, microfluidic patterning, and bioprinting provide programmable control over tissue geometry, which is essential for generating hierarchical and perfusable vascular networks [295]. Despite substantial progress in extrusion‐based bioprinting, recreating multiscale vascular architectures that span capillary, arteriolar, and larger vessel dimensions remain an unresolved challenge. While endothelial lumen formation and localized perfusion have been demonstrated, fully integrated vascular hierarchies capable of sustained function have not yet been achieved. A central question is whether vascularization strategies should primarily follow engineering‐driven reconstruction or instead draw more heavily on principles from developmental biology. Notably, these approaches are not mutually exclusive. Developmental studies indicate that early vascular morphogenesis arises from coordinated angiogenic sprouting, branching, and hierarchical remodeling, offering conceptual guidance for in vitro vascular design. In this context, combining bioprinting with spatially defined angiogenic cues or self‐organizing vascular progenitor populations may provide effective routes toward functional vascularization. Equally important is the incorporation of supporting cell types, including FBs, pericytes, and immune cells which contribute to ECM remodeling, vascular stabilization, and tissue homeostasis.

Integrating multiple bioengineering strategies, rather than optimizing individual components in isolation, is therefore likely to be necessary for translational cardiac tissue engineering. Particular emphasis should be placed on approaches that promote CM maturation while simultaneously optimizing scaffold architecture to establish coherent structure and function relationships, especially in thick cardiac patches and volumetric heart constructs [295, 296]. Achieving synchronized electromechanical coupling between CMs and vascular cells represents an additional barrier, as effective electrical integration is required to support rhythmic contraction and continuous perfusion. Bioreactor platforms that combine mechanical loading, electrical pacing, and controlled perfusion have shown promise in improving CM alignment and functional maturation.

At present, evaluation of vascularized cardiac tissues is complicated by the use of heterogeneous and non‐standardized functional endpoints. Although individual assays provide valuable information, inconsistent metrics across platforms hinder direct comparison and large‐scale data integration. To improve reproducibility, future studies would benefit from adopting a core set of quantitative benchmarks that capture both vascular and myocardial performance. For vascular networks, assessments such as flow velocity and uniformity [297], barrier integrity and selective permeability [298], and long‐term perfusion stability should be considered. For myocardial tissues, synchronized contractility, conduction velocity, calcium handling, and metabolic maturation should be evaluated in conjunction with vascular function rather than independently. Increasingly, artificial intelligence (AI)‐based tools are being incorporated into tissue engineering workflows to enable automated monitoring of tissue structure and function. By integrating imaging, electrophysiological, and molecular datasets, AI‐driven systems can support early quality control, longitudinal assessment, and cross‐platform standardization. Such data‐centric frameworks may facilitate regulatory alignment and accelerate the translation of vascularized cardiac tissues toward clinically and industrially relevant applications. Nonetheless, beyond the technical integration of perfusable vasculature, significant system‐level challenges remain, including scalability, batch‐to‐batch reproducibility, long‐term functional stability, and immunological safety.

Beyond the successful integration of perfusable vasculature into thick and large‐scale cardiac tissues, the translation of vascularized myocardial constructs toward clinically and industrially relevant platforms inevitably faces a set of interconnected, system‐level challenges. These include scale‐up limitations, batch‐to‐batch reproducibility, long‐term functional stability, as well as immunogenicity and safety considerations. Importantly, addressing these system‐level constraints is closely coupled to the expanding application landscape enabled by perfusable cardiac tissues. Once perfusable vasculature is successfully integrated into thick and large‐scale cardiac tissues, future applications of vascularized cardiac constructs may expand to scenarios in clinical applications. Vascularized cardiac tissues provide a unique platform for investigating vascular‐related cardiac diseases, such as MI, coronary microvascular dysfunction, and diabetic cardiomyopathy. For example, compared with the traditional in vitro cardiac models to recapitulate MI, the perfusable vasculature and dynamic ischemia/reperfusion system may replicate key pathological processes such as microvascular obstruction and flow‐dependent tissue injury. Additionally, coronary microvascular dysfunction could only be modelled in vitro when existing capillary‐scale perfusable networks and flow‐regulated endothelial function, to capture microvascular spasm, impaired vasoreactivity, and perfusion‐metabolism mismatch. Beyond single‐organ applications, vascularized myocardial constructs can serve as key modules in multi‐organ and metabolic interaction studies within the MPS. By incorporating patient‐specific cells and adjustable cellular compositions, inter‐organ signaling, systemic drug responses, and disease comorbidities could be modelled. Such capabilities open new avenues for studying whole‐body metabolism, cardiotoxicity, and personalized therapeutic responses, while simultaneously reinforcing the need for standardized manufacturing, functional benchmarking, and safety assessment frameworks to support their broader translational deployment.

## Conflicts of Interest

The authors declare no conflicts of interest.

## Data Availability

Data sharing not applicable to this article as no datasets were generated or analysed during the current study.
